# Reappraisal of Non-vitamin K Antagonist Oral Anticoagulants in Atrial Fibrillation Patients: A Systematic Review and Meta-Analysis

**DOI:** 10.3389/fcvm.2021.757188

**Published:** 2021-10-15

**Authors:** Fuwei Liu, Yunyao Yang, Winglam Cheng, Jianyong Ma, Wengen Zhu

**Affiliations:** ^1^Department of Cardiology, The Affiliated Ganzhou Hospital of Nanchang University, Ganzhou, China; ^2^Department of Cardiology, The First Affiliated Hospital of Sun Yat-Sen University, Guangzhou, China; ^3^Department of Pharmacology and Systems Physiology, University of Cincinnati College of Medicine, Cincinnati, OH, United States

**Keywords:** anticoagulants, atrial fibrillation, propensity score, outcomes, meta-analysis

## Abstract

**Background:** Recent observational studies have compared effectiveness and safety profiles between non-vitamin K antagonist oral anticoagulants (NOACs) and warfarin in patients with atrial fibrillation (AF). Nevertheless, the confounders may exist due to the nature of clinical practice-based data, thus potentially influencing the reliability of results. This systematic review and meta-analysis were conducted to compare the effect of NOACs with warfarin based on the propensity score-based observational studies vs. randomized clinical trials (RCTs).

**Methods:** Articles included were systematically searched from the PubMed and EMBASE databases until March 2021 to obtain relevant studies. The primary outcomes were stroke or systemic embolism (SSE) and major bleeding. Hazard ratios (HRs) and 95% confidence intervals (CIs) of the outcomes were extracted and then pooled by the random-effects model.

**Results:** A total of 20 propensity score-based observational studies and 4 RCTs were included. Compared with warfarin, dabigatran (HR, 0.82 [95% CI, 0.71–0.96]), rivaroxaban (HR, 0.80 [95% CI, 0.75–0.85]), apixaban (HR, 0.75 [95% CI, 0.65–0.86]), and edoxaban (HR, 0.71 [95% CI, 0.60–0.83]) were associated with a reduced risk of stroke or systemic embolism, whereas dabigatran (HR, 0.76 [95% CI, 0.65–0.87]), apixaban (HR, 0.61 [95% CI, 0.56–0.67]), and edoxaban (HR, 0.58 [95% CI, 0.45–0.74]) but not rivaroxaban (HR, 0.92 [95% CI, 0.84–1.00]) were significantly associated with a decreased risk of major bleeding based on the observational studies. Furthermore, the risk of major bleeding with dabigatran 150 mg was significantly lower in observational studies than that in the RE-LY trial, whereas the pooled results of observational studies were similar to the data from the corresponding RCTs in other comparisons.

**Conclusion:** Data from propensity score-based observational studies and NOAC trials consistently suggest that the use of four individual NOACs is non-inferior to warfarin for stroke prevention in AF patients.

## Introduction

Atrial fibrillation (AF), the most common arrhythmia in clinical practice, increases the five-fold risk of ischemic stroke and two-fold for all-cause mortality (1, 2). Before 2010, warfarin was primarily used to prevent stroke in AF patients, but there is a limited range for treatment due to the regular monitoring of the international normalized ratio (INR), and the dosage is adjusted frequently (3). Subsequently, non-vitamin K oral anticoagulants (NOACs), including direct thrombin inhibitor (dabigatran) and factor Xa inhibitors (rivaroxaban, apixaban, and edoxaban) are recommended as the preferred drugs for stroke prevention among nonvalvular AF patients (4–6). Compared with warfarin, NOACs do not require anticoagulation monitoring, have easier dosing regimens, and have fewer food and drug interactions (7).

Previous randomized clinical trials (RCTs) have shown that the efficacy and safety of the NOACs are superior or non-inferior to warfarin in AF patients. Specifically, compared with warfarin, dabigatran is associated with lower rates of stroke and systemic embolism (SSE) and a similar rate of major bleeding (8), apixaban has decreased rates of SSE and MB (9), rivaroxaban has non-inferior rates of SSE and a similar rate of major bleeding (10), and edoxaban has non-inferior rates of SSE and a lower rate of major bleeding (11). Although RCTs could ensure the balance of results between different patient groups and get a fair evaluation of the trial treatment effect, they limit the assessment of the risks and benefits of interventions for all the populations when these interventions are used in real-world settings. By contrast, observational studies could infer a wider range of patient characteristics and evaluate a broader range of outcomes over a more extended period (12, 13). More recently, many observational studies have been published to compare the effectiveness and safety of NOACs vs. warfarin in AF patients. However, the obvious confounders and significant biases may exist in several observational studies due to the nature of clinical practice-based data, thus potentially influencing the reliability of findings.

An effective method to evaluate interventions' effectiveness in typical clinical settings can be provided by the propensity score (PS) (14). Observational studies using the PS method may alter the target population by changing the distribution of patient baseline characteristics that facilitate analysis. Therefore, the PS analysis can be used to reduce biases in comparisons between the targeted populations and controls. In the present meta-analysis, we aimed to compare the effectiveness and safety profiles between NOACs and warfarin based on the PS-based observational studies, and further test whether the pooled results of high-quality observational studies were consistent with data from the corresponding RCTs.

## Methods

This systematic review and meta-analysis were carried out based on the Cochrane Handbook for systemic reviews. The results were presented according to the Preferred Reporting Items for Systematic Reviews and Meta-Analyses (PRISMA) Statement. Ethical approval was not provided because we only included the published studies.

We performed a systematic search in detail on the PubMed and EMBASE databases until March 2021 to obtain all the relevant studies. To obtain a balanced covariate distribution between groups of NOACs and warfarin, we included observational articles that applied the PS-based methods. In addition, 4 RCTs of NOACs vs. warfarin were also selected (dabigatran [RE-LY], rivaroxaban [ROCKET AF], apixaban [ARISTOTLE], and edoxaban [ENGAGE AF-TIMI 48]). The primary outcomes were SSE and major bleeding. Data extraction was conducted independently by two researchers. The hazard ratios (HR) and 95% confidence intervals (CIs) were considered as the effect sizes, and the pooled by the random-effects model. To test the stability of the results, we re-conducted the analysis using the fixed-effects model, inverse variance heterogeneity (IVhet), and quality effects (QE) models. Detailed information including eligibility criteria, literature search, study selection, and data extraction, quality assessment, and statistical analysis was provided in Supplementary Materials.

All the statistical analyses were carried out by Review Manager 5.3 software (the Cochrane Collaboration 2014. Nordic Cochrane Centre Copenhagen, Denmark), the Stata software (version 16.0, Stata Corp LP, College Station, TX), and MetaXL (version 5.3).

## Results

### Study Selection

The flow chart of document retrieval is presented in Supplementary Figure 1. A total of 1,139 studies from two electronic databases were under-identification. A total of 782 studies remained after duplication removal, and then 57 studies were left based on the screenings of titles/abstracts. Among the 57 studies undergoing the full-text screenings, 33 of them were excluded due to the following reasons: (1) 23 studies used overlapping databases; (2) 3 studies included single-center patients, and the sample size was less than 1,000; (3) 5 studies reported the comparisons between combined NOACs vs. warfarin, or did not regard warfarin as the reference; (4) 2 studies did not use the PS-based methods to match baseline patient characteristics. Finally, 24 studies (3, 7–11, 15–32) (20 observational cohort studies and 4 RCTs) were included in our current meta-analysis.

### Baseline Characteristics of the Included Studies

The baseline characteristics of the included RCTs are shown in Supplementary Table 1. Detailed information was categorized into different groups based on the dose of NOACs. Baseline characteristics of the 20 observational studies are shown in Table 1. Although some studies extracted data from the same database, they analyzed different kinds of NOACs, included diverse study periods, or included different outcomes for analysis. For instance, both Gupta et al. (25) and Villines et al. (26) obtained data from the US Department of Defense, but the study periods ranged from 2013 to 2015 for Gupta et al., and from 2009 to 2012 for Villines et al. All the included studies applied the PS-based methods to balance the covariates between groups [propensity score matching [PSM], *n* = 11 (3, 7, 20, 23–27, 29, 31, 32), and inverse probability of treatment weighting [IPTW], *n* = 9 (15–19, 21, 22, 28, 30)]. For the PS diagnostics, 14 studies used standardized differences, and 6 studies failed to report any further diagnostic use.

**Table 1:** Baseline characteristics of included observational studies.

**Included studies**	**Location**	**Data source**	**Comparisons**	Sample size (n)^*^	Age (y)^*^	Female (%)^*^	Follow-up (months)^*^	**Outcomes in the analysis**	**PS methods**
Mitsuntisuk et al. (15)	Thailand	REAL-T AF trial, 01/2012–04/2018; age≥18 years; retrospective	DA vs. WAR RIV vs. WAR API vs. WAR	405/605 441/605 604/605	71.63/68.40	48.21/50.25	26.44/33.84	SSE, MB, IS, all-cause death, ICH, GIB	IPTW
Nielsen et al. (16)	Denmark	Three Danish nationwide databases, 08/2011–02/2016; retrospective	DA vs. WAR RIV vs. WAR API vs. WAR	4400/38893 8875/38893 3476/38893	80.54/71.00	55.41/40.40	27.60	SSE, MB, IS, all-cause death	IPTW
Larsen et al. (17)	Denmark	Three Danish nationwide database, 08/2011–10/2015; retrospective	DA vs. WAR RIV vs. WAR API vs. WAR	6349/35436 12701/35436 7192/35436	69.65/72.40	37.82/41.20	22.80	SSE, MB, IS, all cause-death, ICH	IPTW
Kohsaka et al. (18)	Japan	MDV, 03/2011–07/2018, retrospective	DA vs. WAR RIV vs. WAR API vs. WAR EDO vs. WAR	22752/19059 8003/19059 12592/19059 17481/19059	76.08/76.10	38.74/38.80	24.00	SSE, MB, IS, ICH, GIB	IPTW
Lee et al. (19)	Korea	Korean Health Insurance Review and Assessment database, 01/2015–12/2017, retrospective	DA vs. WAR RIV vs. WAR API vs. WAR EDO vs. WAR	35965/25420 17745/25420 22177/25420 15496/25420	70.93/71.20	44.36/45.50	-	MB, IS, ICH, GIB	IPTW
Cha et al. (20)	Korea	NHIS, 01/2014–12/2015, retrospective	DA vs. WAR RIV vs. WAR API vs. WAR	5681/23222 3741/23222 2189/23222	70.08/68.82	45.27/43.10	5.97/18.12	IS, all-cause death, ICH	PSM
Bang et al. (21)	Korea	Korea's nationwide health insurance claims database, 01/2015–11/2016, retrospective	DA vs. WAR RIV vs. WAR API vs. WAR	-	-	-	-	SSE, MB, ICH, GIB	IPTW
Chan.et al. (22)	Taiwan	Taiwan's National Health Insurance Research Database, 06/2012–12/2017, retrospective	DA vs. WAR RIV vs. WAR API vs. WAR EDO vs. WAR	4577/19761 9952/19761 33022/19761 22371/19761	74.7/74.6 74.8/74.6 74.7/74.6 74.7/74.6	42.8/43.3 42.4/43.3 42.5/43.3 42.6/43.3	16	SSE, MB, IS, ICH, GIB	IPTW
Laliberte et al. (23)	USA	SHS Patient Transactional Datasets, 05/2011–07/2012, retrospective	RIV vs. WAR	3654/14616	73.30/73.70	51.00/51.50	2.77/3.77	SSE, MB, IS, ICH, GIB	PSM
Wanat et al. (24)	USA	GE Centricity EMR database, 01/2012–12/2016, retrospective	API vs. WAR	10189/10189	72.10/72.20	46.90/46.60	12.00	SSE	PSM
Gupta et al. (25)	USA	DOD, 01/01/2013–30/09/2015, retrospective	DA vs. WAR RIVvs. WAR API vs. WAR	3691/3691 8226/8226 7607/7607	76.03/76.07	41.31/41.20	5.60/5.03	SSE, MB, IS, ICH, GIB	PSM
Villines et al. (26)	USA	DOD, 10/2009–07/2012, retrospective	DA vs. WAR	12793/12793	73.80/74.00	41.20/41.10	9.91/7.24	MB, IS, all-cause death, ICH, GIB	PSM
Russo-Alvarez et al. (27)	USA	CCHS, 01/2012–07/2016, retrospective	RIV vs. WAR	472/472	73.60/73.60	38.80/36.40	-	MB	PSM
Adeboyeje et al. (28)	USA	HIRE, 11/2010–02/2015, retrospective	DA vs. WAR RIVvs. WAR API vs. WAR	8539/23431 3689/23431 8398/23431	70.00/70.00	41.07/40.90	6.05/9.50	MB, ICH, GIB	IPTW
Chang et al. (29)	USA	IMS Health LifeLink Health Plan Claims Database, 10/2010–03/2012, retrospective	DA vs. WAR RIV vs. WAR	4907/39607 1649/39607	60.89/57.40	36.08/46.90	1.95/1.57	GIB	PSM
Lip et al. (3)	USA	US Centers for Medicare and Medicaid Services Medicare data and 4 commercial claims database, 01/01/2013–30/09/2015retrospective	DA vs. WAR RIV vs. WAR API vs. WAR	100977/100977 36990/36990 125068/125068	75.45/75.48	47.11/47.00	4.51/5.27	SSE, MB, IS, ICH, GIB	PSM
Hernandez et al. (30)	USA	CMS, 10/2010–10/2011, retrospective	DA vs. WAR	1302/8102	75.10/75.60	57.90/59.00	5.90/7.60	MB, ICH, GIB	IPTW
Huybrechts et al. (31)	USA	MarketScan and Optum, 10/2010–09/2015, prospective	DA vs. WAR RIV vs. WAR API vs. WAR	29448/29448 35520/35520 19588/19588	69.88/69.78	38.95/38.42	-	SSE, MB, IS, all-cause death, ICH, GIB	PSM
Bradley et al. (7)	USA	SDD, 12/2012–06/2018, age≥21 years, retrospective	API vs. WAR	55038/55030	71.30/71.30	39.30/39.20	-	IS, ICH, GIB	PSM
Go et al. (32)	USA	SDD, 11/2010–05/2014, age≥21 years, retrospective	DA vs. WAR	25289/25289	68.48.68.34	36.10/35.70	4.10/3.40	IS, ICH, GIB	PSM

* *Data after PSM or IPTW*.

*MDV, Medical Data Vision Co Ltd; NHIS, Korean National Health Insurance Service database; SHS, Symphony Health Solutions' (SHS) Patient Transactional Datasets; DOD, US Department of Defense; CCHS, Cleveland Clinic Health System; HIRE, HealthCore Integrated Research Environment; CMS, Centers for Medicare and Medicaid Services; SDD, the Sentinel Distributed Database;DA, dabigatran; RIV, rivaroxaban; API, apixaban; EDO, edoxaban; WAR, warfarin; SSE, stroke or systemic embolism; MB, major bleeding; IS, ischemic stroke; ICH, intracranial hemorrhage; GIB, gastrointestinal bleeding; PS, Propensity Score; PSM, propensity score matching; IPTW, inverse probability of treatment weighting; NA, diagnostic not available; SD, standardized difference*.

The results of the risk of bias assessment for RCTs are shown in Supplementary Table 2, suggesting low risks in biases. The methodological quality assessment of observational cohorts was carried out by the NOS tool (Supplementary Table 3). All articles scored 7 or more points indicating relatively high quality.

### Comparisons Between Individual NOAC and Warfarin

Based on the observational studies, the crude event rates and pooled HRs (based on random-effects model) of the outcomes between each NOAC vs. warfarin are summarized in Table 2.

**Table 2:** Pooled HRs of the effectiveness and safety outcomes between NOACs vs. warfarin in patients with AF.

	**SSE**	**Major bleeding**	**Ischemic stroke**	**All-cause death**	**Intracranial hemorrhage**	**Gastrointestinal bleeding**
**DA vs. WAR**						
No. of effect estimates	9	13	11	5	13	12
Crude event rates	2.08 vs. 2.89%	2.65 vs. 4.14%	1.46 vs. 2.14%	4.34 vs. 8.55%	0.29 vs. 0.81%	1.26 vs. 1.57%
HRs and 95% CIs	0.82 (0.71–0.96)	0.76 (0.65–0.87)	0.93 (0.86–1.00)	0.75 (0.53–1.04)	0.46 (0.38–0.55)	0.97 (0.80–1.17)
*P*-value	0.01	0.0001	0.06	0.08	<0.00001	0.73
I^2^ statistic	82%	91%	25%	91%	66%	93%
**RIV vs. WAR**						
No. of effect estimates	10	13	10	4	11	10
Crude event rates	1.37 vs. 2.29%	3.31 vs. 4.14%	1.36 vs. 2.18%	8.60 vs. 11.69%	0.47 vs. 0.89%	1.72 vs. 1.83%
HRs and 95% CIs	0.80 (0.75–0.85)	0.92 (0.84–1.00)	0.84 (0.79–0.90)	1.02 (0.77–1.36)	0.69 (0.63,0.76)	0.96 (0.82,1.12)
*P*-value	<0.00001	0.06	<0.00001	0.88	<0.00001	0.62
I^2^ statistic	15%	83%	29%	94%	27%	89%
**API vs. WAR**						
No. of effect estimates	10	11	10	4	11	9
Crude event rates	1.08 vs. 2.47%	2.12 vs. 4.35%	0.85 vs. 1.96%	3.24 vs. 10.41%	0.27 vs. 0.80%	0.78 vs. 1.73%
HRs and 95% CIs	0.75 (0.65–0.86)	0.61 (0.56–0.67)	0.73 (0.62–0.86)	0.77 (0.39–1.54)	0.62 (0.50–0.75)	0.63 (0.54–0.73)
*P*-value	<0.0001	<0.00001	0.0002	0.46	<0.00001	<0.00001
I^2^ statistic	88%	73%	83%	97%	75%	84%
**EDO vs. WAR**						
No. of effect estimates	2	3	3	-	2	3
Crude event rates	1.16 vs. 3.84%	0.88 vs. 2.80%	1.17 vs. 2.83%	-	0.22 vs. 1.10%	0.62 vs. 1.66%
HRs and 95% CIs	0.71 (0.60–0.83)	0.58 (0.45–0.74)	0.67 (0.59–0.76)	-	0.60 (0.25–1.44)	0.65 (0.41–1.04)
*P*-value	<0.0001	<0.0001	<0.00001	-	0.25	0.07
I^2^ statistic	0%	68%	0%	-	95%	90%

*SSE, stroke or systemic embolism; DA, dabigatran; RIV, rivaroxaban; API, apixaban; EDO, edoxaban; WAR, warfarin; HR, hazard ratio; CI, confidence interval*.

#### Primary Outcomes Between Each NOAC vs. Warfarin

**Figure 1:**
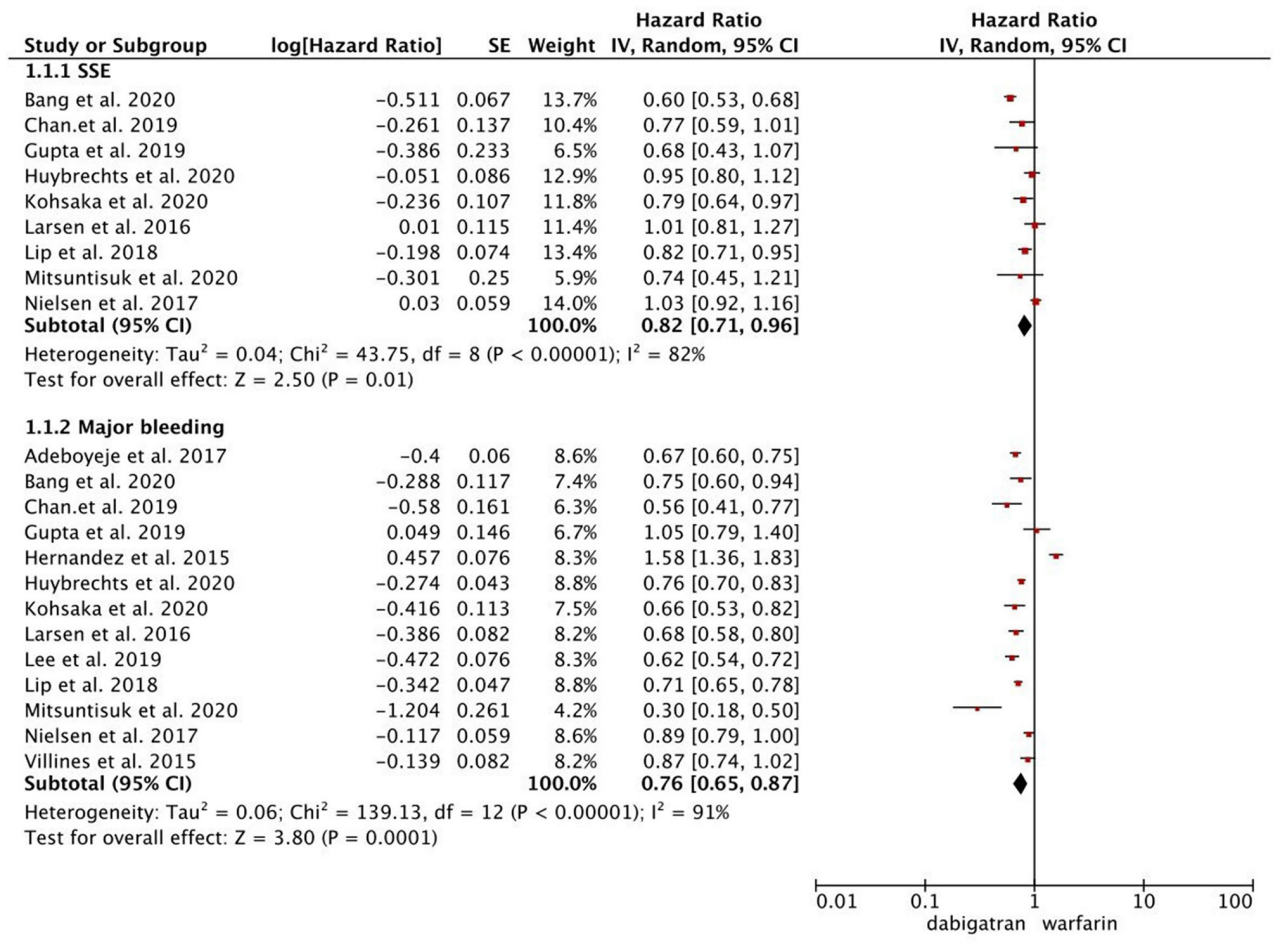
Comparing the primary outcomes including SSE and MB of dabigatran vs. warfarin. SSE, stroke or systemic embolism; MB, major bleeding; HR, hazard ratio; CI, confidence interval; SE, standard error; IV, inverse of the variance.

**Figure 2:**
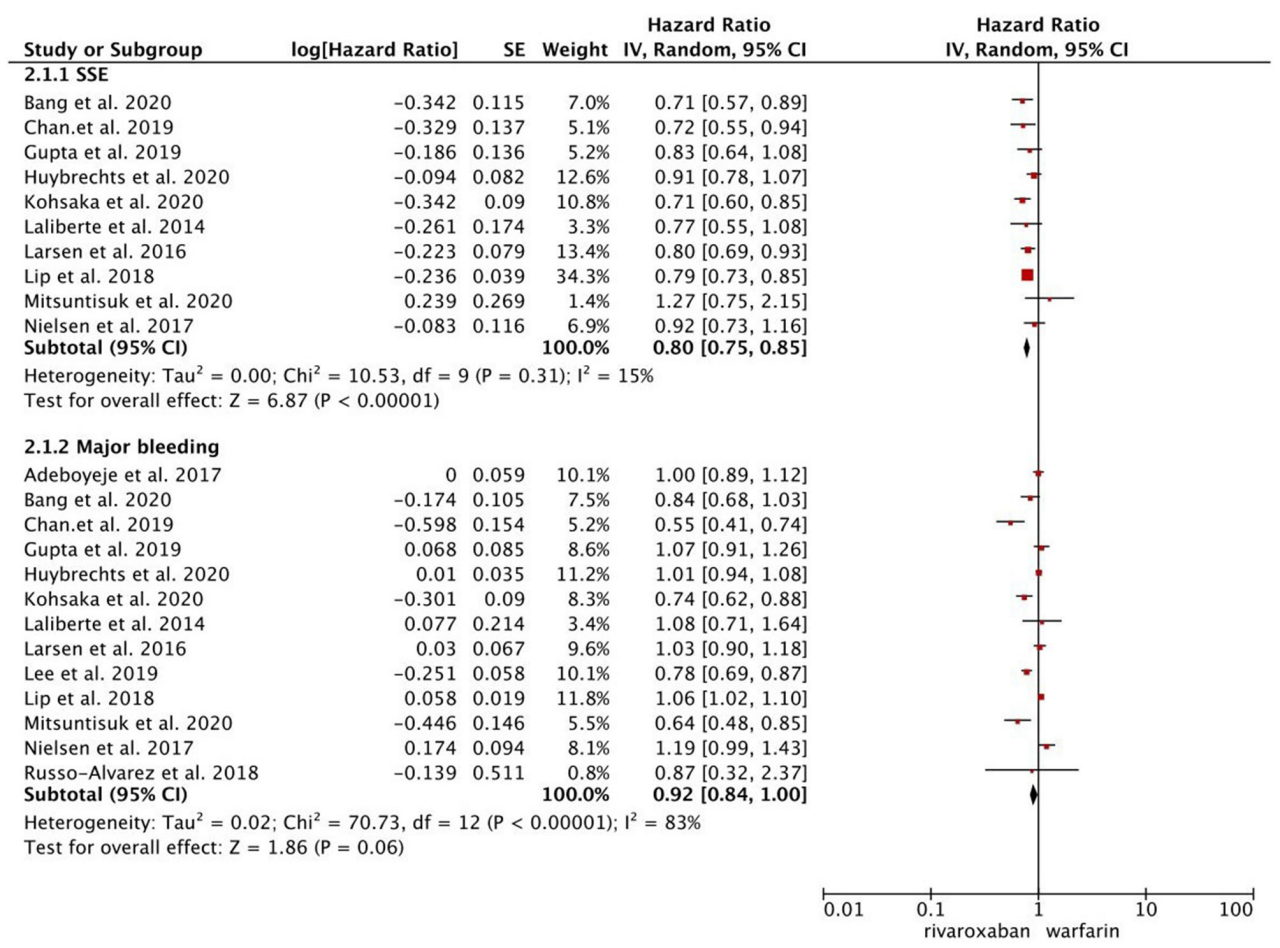
Comparing the primary outcomes including SSE and MB of rivaroxaban vs. warfarin. SSE, stroke or systemic embolism; MB, major bleeding; HR, hazard ratio; CI, confidence interval; SE, standard error; IV, inverse of the variance.

**Figure 3:**
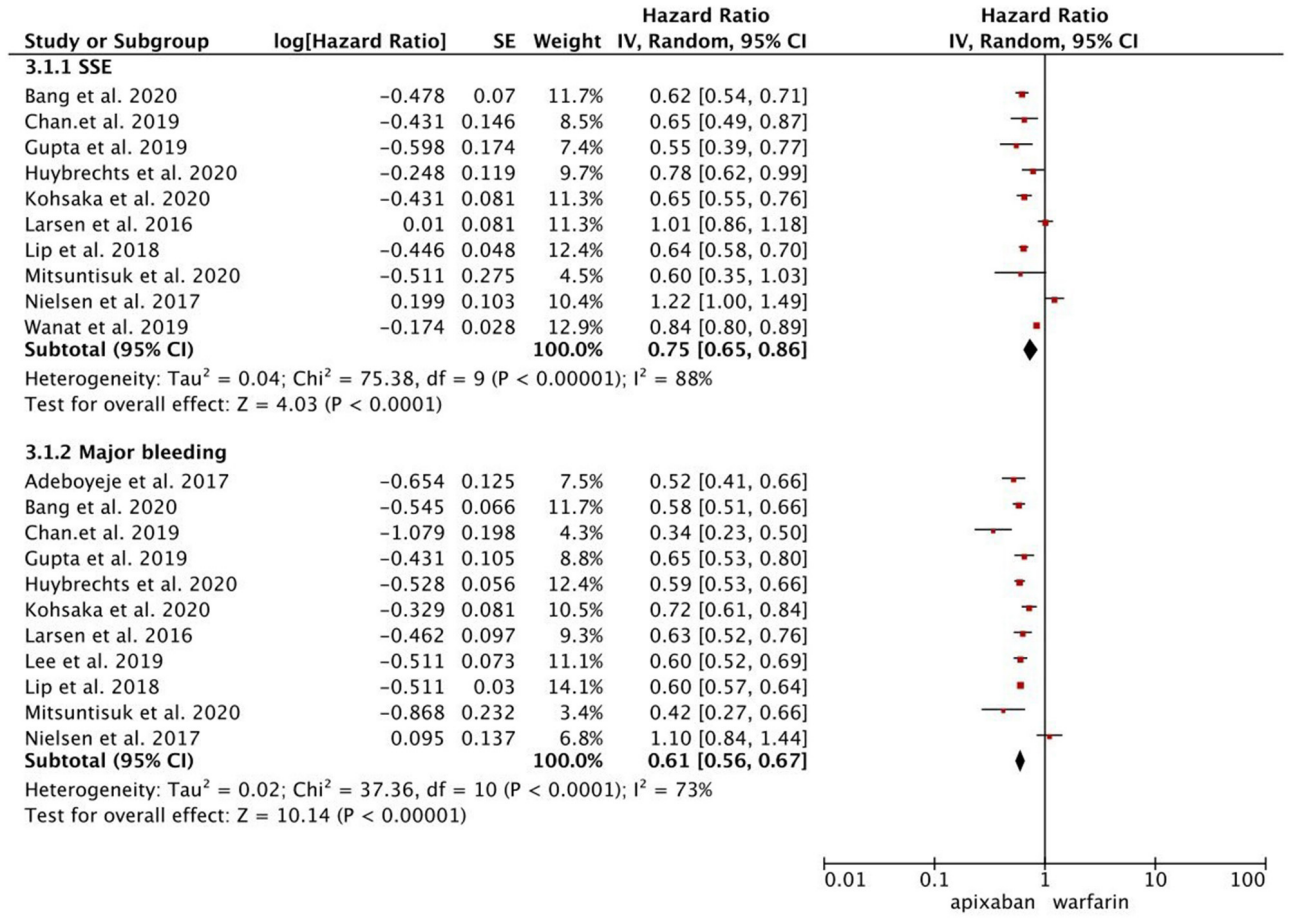
Comparing the primary outcomes including SSE and MB of apixaban vs. warfarin. SSE, stroke or systemic embolism; MB, major bleeding; HR, hazard ratio; CI, confidence interval; SE, standard error; IV, inverse of the variance.

As presented in Figure 1, compared with warfarin, dabigatran was associated with reduced risks of SSE (2.08 vs. 2.89%; HR, 0.82 [95% CI, 0.71–0.96]) and major bleeding (2.65 vs. 4.14%; HR, 0.76 [95% CI, 0.65–0.87]). The results of rivaroxaban vs. warfarin are shown in Figure 2. Compared with warfarin use, the use of rivaroxaban was markedly associated with a reduced risk of SSE (1.37 vs. 2.29%; HR, 0.80 [95% CI, 0.75–0.85]). Meanwhile, it presented a comparable risk of major bleeding (3.31 vs. 4.14%; HR, 0.92 [95% CI, 0.84–1.00]) between rivaroxaban vs. warfarin. As shown in Figure 3, the use of apixaban vs. warfarin was related to reduced risks of SSE (1.08 vs. 2.47%; HR, 0.75 [95% CI, 0.65–0.86]) and major bleeding (2.12 vs. 4.35%; HR, 0.61 [95% CI, 0.56–0.67]). As shown in Supplementary Figure 2, compared with warfarin use, the use of edoxaban was significantly associated with decreased risks of SSE (1.16 vs. 3.84%; HR, 0.71 [95% CI, 0.60–0.83]) and major bleeding (0.88 vs. 2.80%; HR, 0.58 [95% CI, 0.45–0.74]).

#### Secondary Outcomes Between NOAC vs. Warfarin

Compared with warfarin, dabigatran was associated with reduced risks of ischemic stroke (HR, 0.93 [95% CI, 0.86–1.00]) and intracranial hemorrhage (HR, 0.46 [95% CI, 0.38–0.55]), but had similar risks of all-cause death and gastrointestinal bleeding (Supplementary Figure 3). As for rivaroxaban vs. warfarin shown in Supplementary Figure 4, it was associated with reduced risks of ischemic stroke (HR, 0.84 [95% CI, 0.79–0.90]) and intracranial hemorrhage (HR, 0.69 [95% CI, 0.63–0.76]), but had comparable risks of all-cause death and gastrointestinal bleeding. The use of apixaban vs. warfarin was significantly associated with reduced risks of ischemic stroke (HR, 0.73 [95% CI, 0.62–0.86]), intracranial hemorrhage (HR, 0.62 [95% CI, 0.50–0.75]), and gastrointestinal bleeding (HR, 0.63 [95% CI, 0.54–0.73]), but displayed no difference in all-cause death (Supplementary Figure 5). The use of edoxaban vs. warfarin was related to a decreased risk of ischemic stroke (HR, 0.67 [95% CI, 0.59–0.76]), whereas similar risks were observed in intracranial hemorrhage and gastrointestinal bleeding between the two study groups (Supplementary Figure 2).

#### Sensitivity Analysis and Subgroup Analysis

In the sensitivity analysis, the results of the primary outcomes from the IVhet or QE models (Supplementary Figures 6–9) were similar to those from the primary analysis using the random-effects model. In addition, the results did not change substantially when we re-conducted the analyses using the fixed-effects model (Supplementary Table 4).

As shown in Supplementary Table 4, the subgroup analyses concerning the primary outcomes suggested no significant interactions grouped by the NOAC-dose and follow-up period. For the subgroup analysis based on the regions, Asians showed fewer risks of SSE and major bleeding than non-Asians in the group of dabigatran vs. warfarin. In the group of rivaroxaban vs. warfarin, Asians showed fewer risks of major bleeding compared with non-Asians. In the group of apixaban vs. warfarin, the risk of SSE was significantly lowered in Asians compared with non-Asians. There were not enough studies for the subgroup analyses between edoxaban vs. warfarin.

### Summary Effect Estimates Between Observational Studies and RCTs

Comparative effect estimates of NOACs vs. warfarin between observational studies and RCTs are shown in Table 3. For the primary outcomes, dabigatran 150 mg vs. warfarin had a significantly lower risk of major bleeding in the observational studies (HR, 0.72 [95% CI, 0.66–0.78]) than that in the RE-LY trial (HR, 0.93 [95% CI, 0.81–1.07]) (*P*_interaction_ = 0.002). In other comparisons, the pooled effects of the observational studies were consistent with data from the corresponding NOAC trials.

**Table 3:** Comparing total effect estimates of NOACs vs. warfarin between observational studies and RCTs.

	**Dabigatran vs. Warfarin**				**Rivaroxaban vs. Warfarin**		**Apixaban vs. Warfarin**		**Edoxaban vs. Warfarin**	
	**Dabigatran 110 mg**		**Dabigatran 150 mg**		**Rivaroxaban 15/20 mg**		**Apixaban 2.5/5 mg**		**Edoxaban 60 mg**	
	**HR (95% CI)**	***P***	**HR (95% CI)**	***P***	**HR (95% CI)**	***P***	**HR (95% CI)**	***P***	**HR (95% CI)**	***P***
**Primary outcomes SSE**										
Observational	0.94 (0.76,1.16)	0.83	0.82 (0.68,0.98)	0.14	0.78 (0.72,0.84)	0.18	0.68 (0.54,0.84)	0.29	0.76 (0.39,1.47)	0.72
RCT^*^	0.91 (0.74,1.11)		0.66 (0.53,0.82)		0.88 (0.74,1.03)		0.79 (0.66,0.95)		0.86 (0.74,1.01)	
**MB**										
Observational	0.77 (0.53,1.10)	0.83	0.72 (0.66,0.78)	0.002	1.07 (1.03,1.13)	0.70	0.59 (0.51,0.67)	0.11	0.81 (0.15,4.39)	0.99
RCT^*^	0.80 (0.69,0.93)		0.93 (0.81,1.07)		1.04 (0.90,1.20)		0.69 (0.60,0.80)		0.80 (0.71,0.91)	
**Secondary outcomes IS**										
Observational	0.96 (0.77,1.21)	0.39	0.99 (0.83,1.20)	0.009	0.91 (0.79,1.04)	0.8	0.72 (0.57,0.91)	0.21	-	-
RCT^*^	1.11 (0.89,1.40)		0.76 (0.60,0.98)		0.94 (0.75,1.17)		0.92 (0.74,1.34)		-	
**All-cause death**										
Observational	1.05 (0.99,1.12)	0.04	0.65 (0.54,0.79)	0.05	1.19 (0.78,1.80)	0.15	0.77 (0.39,1.54)	0.69	-	-
RCT^*^	0.91 (0.80,1.03)		0.88 (0.77,1.00)		0.85 (0.70,1.03)		0.89 (0.80,1.00)		-	
**ICH**										
Observational	0.51 (0.30,0.86)	0.15	0.43 (0.33,0.57)	0.77	0.61 (0.47,0.81)	0.7	0.61 (0.45,0.81)	0.1	-	-
RCT^*^	0.31 (0.20,0.47)		0.40 (0.27,0.60)		0.67 (0.47,0.93)		0.42 (0.30,0.58)		-	
**GIB**										
Observational	0.77 (0.49,1.21)	0.18	1.03 (0.83,1.28)	0.02	1.29 (1.05,1.58)	0.45	0.58 (0.43,0.77)	0.03	-	-
RCT^*^	1.10 (0.86,1.41)		1.50 (1.19,1.89)		1.42 (1.22,1.66)		0.89 (0.70,1.15)		-	

* *Corresponding RCTs for the dabigatran group, rivaroxaban group, apixaban group and edoxaban group are RE-LY (8), ROCKET-AF (10), ARISTOTLE (9) and ENGAGE AF-TIMI 48 [11], respectively*.

*NOAC, non-vitamin K antagonist oral anticoagulant; RCT, randomized controlled trials; HR, hazard ratio; CI, confidence interval; SSE, stroke or systemic embolism; MB, major bleeding; IS, ischemic stroke; ICH, intracranial hemorrhage; GIB, gastrointestinal bleeding*.

For the secondary outcomes, dabigatran 110 mg vs. warfarin demonstrated a higher risk of all-cause death in observational studies (HR, 1.05 [95% CI, 0.99–1.12]) than that in the RE-LY trial (HR, 0.91 [95% CI, 0.80–1.03]) (*P*_interaction_ = 0.04). Dabigatran 150 mg vs. warfarin showed a lower risk of gastrointestinal bleeding in observational studies (HR, 1.03 [95% CI, 0.83–1.28]) compared with that in the RE-LY trial (HR, 1.50 [95% CI, 1.19–1.89]) (*P*_interaction_ = 0.02). The pooled HR of apixaban 5/2.5 mg vs. warfarin for gastrointestinal bleeding was significantly lower in observational studies (HR, 0.58 [95% CI, 0.43–0.77]) compared to that of the ARISTOTLE trial (HR, 0.89 [95% CI, 0.70–1.15]) (*P*_interaction_ = 0.03). Meanwhile, all the effect estimates of rivaroxaban vs. warfarin were similar between observational studies and the ROCKET AF trial, whereas no enough studies assessed the secondary outcomes of edoxaban vs. warfarin between observational studies and the ENGAGE AF-TIMI 48 trial.

### Publication Bias

For the observational studies, there were no potential publication biases when inspecting the funnel plots of the primary outcomes (Supplementary Figures 10–13). In addition, the Begg's and Egger's tests also proved no significant publication biases (all *P* > 0.1; Supplementary Table 5). For the secondary outcomes, the Egger's test showed a potential publication bias in intracranial hemorrhage of the dabigatran vs. warfarin group, and ischemic stroke of the rivaroxaban vs. warfarin group. Nevertheless, the results from the trim-and-fill analysis suggested no trimming performed, and the corresponding pooled results were not changed. For the RCTs, there was no need for publication bias analysis because only four NOAC trials were included.

## Discussion

In the current meta-analysis, we compared the studied outcomes between NOACs and warfarin by only included the PS-based observational studies. Based on the observational studies, the results from different pooled models consistently suggested that compared with warfarin, dabigatran, rivaroxaban, apixaban, and edoxaban were associated with a reduced risk of SSE, whereas dabigatran, apixaban, and edoxaban but not rivaroxaban was associated with a decreased risk of major bleeding. We further tested whether the pooled results of high-quality observational studies were consistent with data from the corresponding RCTs. The risk of major bleeding with dabigatran 150 mg was significantly lower in observational studies than that in the RE-LY trial, whereas the pooled results of observational studies were consistent with data from the corresponding RCTs in other comparisons for both SSE and major bleeding.

Over the past few decades, vitamin K antagonists such as warfarin have been confirmed to be effective for preventing stroke in AF patients (33). However, the shortcomings of warfarin mainly include slow onset time, the significantly varied dose-response relationship among patients, narrow therapeutic window, and frequent interactions with other drugs, potentially limiting its clinical applications (34). Nowadays, there is increasing use of NOACs because they could be more effective, easier to control, and safer than warfarin (7). Previous NOAC trials (RE-LY, ROCKET-AF, ARISTOTLE, and ENGAGE-AF TIMI 48) suggested that NOACs were comparable to warfarin in efficacy, but NOACs significantly reduced the risk of bleeding. Based on data of NOAC trials, current guidelines have recommended NOACs as the first-line drugs for the prevention of thrombogenesis and stroke in patients with nonvalvular AF (5). Although, RCTs have always been hailed as the gold standard for clinical efficacy evaluation, their results may not be well applicable in practice. At this time, observational studies can be a useful complement (35).

Nowadays, clinical practice-based data are increasingly used to evaluate the effectiveness and safety profiles of NOACs compared to warfarin. Xue et al. (34) compared the overall effectiveness and safety outcomes of three NOACs (dabigatran, rivaroxaban, and apixaban) with warfarin in Asians with AF. Based on the real-world studies, the authors demonstrated that in Asians with AF, the use of NOACs could have potential advantages in all the effectiveness and safety profiles when compared to warfarin irrespective of the type and drug doses. Nevertheless, the heterogeneous real-world studies without proper methods to balance the covariate distribution could be influenced by the potential confounders (36), thus potentially influencing the reliability of results. The PS methods including PSM and IPTW are the most frequently used methods to deal with this issue. The PS methods comprehensively consider all measured characteristic variables, especially confounding factors, making the matched sample more similar to the population of an RCT. PSM can match the treatment and non-treatment group based on the PS from low to high, and thus it can control multiple confounders at the same time by only using the matching of PS (37). IPTW is capable of eliminating confounders by conforming to the distribution of PS in each group (37). However, PSM and IPTW are often failed to be properly conducted (36). Therefore, to further improve the reliability of the study outcomes and reduce the influence of confounding factors, PS diagnostics such as standardized differences, C-statistic, and eye-balling could be conducted after PSM or IPTW. Standardized differences are an attribute of the sample, independent of the sample size. It is easy to compute and understand and is the most commonly used diagnostic method to measure the balance of covariate distribution between treatment groups (36, 38). In our current analysis, all of the 20 observational studies applied PSM or IPTW to balance the covariates between NOACs and warfarin regimen group. For the PS diagnostics, 14 studies used standardized differences, and 6 studies failed to report any further diagnostic use.

Reaching an agreement between RCTs and observational studies can greatly improve the accuracy of the results and offer more confidence in the reference of clinical routine practice. It is still known that whether the findings of observational studies were consistent with data from the NOAC trials. Siontis et al. (35) compared the consistency between RCTs and observational studies of the profiles of NOACS and warfarin. The authors found that the effect of NOACs and warfarin were consistent between RCTs and observational studies for most outcomes. However, some exceptions appeared in the dabigatran vs. warfarin group. The RE-LY trial found an increased risk of myocardial infarction in patients treated with dabigatran 150 mg compared with patients using warfarin, whereas the reverse outcomes were found in observational studies. Also, significantly higher risks of major and gastrointestinal bleeding were found in observational studies when compared to the RE-LY trial in the dabigatran group. Conversely, the data of the RE-LY trial demonstrated a lower rate of SSE compared with that of the observational studies. However, Siontis et al. did not describe the baseline characteristics of the treated and non-treated groups in detail, nor did they clarify the statistical methods used in the included studies. Lacking rigorous study design and statistical analysis could make the results easily affected by confounding bias, and thus reduced its reliability. Given these issues, we decided to conduct a more comprehensive meta-analysis by only included the PS-based observational studies. In our analysis, the results of the effectiveness and safety profiles are largely in agreement with some discrepancies that mainly happened in the dabigatran vs. warfarin group. The results of the consistency between the observational studies and RCTs of Siontis et al. are quite similar to our study.

## Limitations

There were still several limitations in this meta-analysis. First, most of the observational studies included were retrospective, and therefore, the association between the drug and the event outcomes rather than their causal relationships were evaluated. Second, despite the detailed information extracted from the included studies, there were still some articles that lack major data (e.g., drug dosage, follow-up period of NOAC treatment) which may provide potential uncertainties to the results. Third, several important cardiovascular events including myocardial infarction were not included in our analysis due to a lack of data. Fourth, in this meta-analysis, we did not include observational studies that only focused on the special populations with AF. Nevertheless, we have previously discussed the effect of NOACs in the special AF populations (e.g., chronic kidney disease, hypertrophic cardiomyopathy, peripheral artery disease, prior stroke) (39–42). Finally, although we included comparisons of outcomes between edoxaban and warfarin, we still failed to assess the results for some outcomes due to insufficient data.

## Conclusion

This meta-analysis suggested that the use of NOACs for stroke prevention in AF was non-inferior or even superior to warfarin based on data from PS-based observational studies. The consistency between the observational studies and corresponding RCTs further confirmed this view.

## Data Availability Statement

The original contributions presented in the study are included in the article/Supplementary Material, further inquiries can be directed to the corresponding author.

## Author Contributions

All authors listed have made a substantial, direct and intellectual contribution to the work, and approved it for publication.

## Funding

This study was funded by National Natural Science Foundation of China (8210020907), China National Postdoctoral Program for Innovative Talents (BX20200400), and China Postdoctoral Science Foundation (2020M673016).

## Conflict of Interest

The authors declare that the research was conducted in the absence of any commercial or financial relationships that could be construed as a potential conflict of interest.

## Publisher's Note

All claims expressed in this article are solely those of the authors and do not necessarily represent those of their affiliated organizations, or those of the publisher, the editors and the reviewers. Any product that may be evaluated in this article, or claim that may be made by its manufacturer, is not guaranteed or endorsed by the publisher.
