# Supplementary material for: Reappraisal of Non-vitamin K Antagonist Oral Anticoagulants in Atrial Fibrillation Patients: A Systematic Review and Meta-Analysis
Source: Front Cardiovasc Med. 2021 Oct 15;8:757188. doi: 10.3389/fcvm.2021.757188 (PMC8554192; doi:10.3389/fcvm.2021.757188)
Supplement: Supplementary file 1 [file Data_Sheet_1.PDF]

Expanded Materials & Methods

This meta-analysis was carried out on the basis of the Cochrane Handbook for systemic reviews[1]. The results of this study were reported according to the Preferred Reporting Items for Systematic Reviews and Meta-Analyses (PRISMA) Statement[2]. Ethical approval was not provided because we only included published studies.

Eligibility criteria

A total of 4 RCTs of NOACs[3-6] (dabigatran[RE-LY; Randomised Evaluation of Long-Term Anticoagulant Therapy], rivaroxaban[ROCKET AF; Rivaroxaban Once Daily Oral Direct Factor Xa Inhibition Compared with Vitamin K Antagonism for Prevention of Stroke and Embolism Trial in Atrial Fibrillation], apixaban[ARISTOTLE; Apixaban for Reduction in Stroke and Other Thromboembolic Events in Atrial Fibrillation] , and edoxaban[ENGAGE AF-TIMI 48; Effective Anticoagulation with Factor Xa Next Generation in Atrial Fibrillation–Thrombolysis in Myocardial Infarction 48]) versus warfarin for stroke prevention in AF patients were included. We attempted to match the propensity score (PS)-based observational studies to RCTs by addressing the similar populations, interventions, comparators, and outcomes. Observational cohort (prospective or retrospective) studies comparing at least one of the effectiveness and/or safety outcomes of NOACs versus warfarin in non-valvular AF patients aged ≥ 18 years were eligible for inclusion. Observational studies would be included if they applied the PS-based methods (e.g., propensity score matching [PSM], stratification, inverse probability of treatment weighting [IPTW], and covariate adjustment) to balance patient characteristics between two treatment groups. We insured that the study populations included were from national or regional registries or registries covering a large population across multiple sites.

Since the common comparator in the four RCTs was warfarin, we excluded observational studies that reported other VKAs (e.g., phenprocoumon and acenocoumarol) or combined VKAs as the reference. Studies that reported combined NOACs were also not included. To maximumly match the study populations in the RCTs, we excluded observational studies that only focused on the specific populations (e.g., elderly, frailty, or AF patients with coronary heart disease, cardiomyopathy, heart failure, chronic kidney disease, hyperthyroidism, cancer). In addition, we also excluded studies restricted to AF patients with certain interventions (e.g., cardioversion, ablation, or left-atrial appendage closure). In order to obtain relatively high-quality articles, single-center studies using local registries were excluded unless they had more than 1000 patients in each group after the PS methods. In addition, cross-sectional studies, case-control studies, reviews, case reports, editorials, letters, or abstracts were excluded. For overlapping studies using the same data sources, the study with the longest follow-up or highest sample size would be selected.

Outcomes

We recorded primary effectiveness outcome as a composite of stroke or systemic embolism (SSE), whereas the primary safety outcome was major bleeding. Secondary effectiveness outcomes were defined as ischemic stroke and all-cause death, whereas the secondary safety outcomes were intracranial hemorrhage and gastrointestinal bleeding.

Some studies would be lack of the primary outcomes because only individual constituents of the composite outcomes were provided. We partly resolved this issue by combining the standardized individual outcomes together to conduct the composite outcome. For instance, Giugliano et al[5] reported the outcomes of stroke and systemic embolism separately, we combined these two effect sizes as the primary outcome of SSE.

Literature search

We did a systematic search in detail on the PubMed and EMBASE databases until March 2021 to obtain all the relevant studies concerning the comparisons between each NOAC versus warfarin in patients with non-valvular AF. The major keywords used in the search strategies were as follows: (1)"atrial fibrillation" OR "atrial flutter" AND (2)"vitamin k antagonists" OR "warfarin" AND (3)"non vitamin k antagonists" OR "direct oral anticoagulants" OR "dabigatran" OR "rivaroxaban" OR "apixaban" OR "edoxaban" AND (4)"propensity" OR "inverse probability". In order to obtain the qualified articles comprehensively, we also did cross-reference retrieval based on the included studies. We considered no language restrictions in the literature research.

Study selection and data extraction

Studies were selected by two researches independently based on the eligibility criteria mentioned above. Qualified articles were included after tittle/abstract screening and full-text screening. Disagreements were resolved through discussion between the two researchers, or consultation with a third researcher.

Data from each included article were extracted by two researchers independently. We extracted information including baseline characteristics of the included studies (author, year of publication, location, data source, sample size, age, percent of female, follow-up period, weight, body mass index, CHADS2 score, comorbidities, medications, TTR of warfarin users,

and INR targets for warfarin), information of NOACs (type, comparisons, dosage), methods to address confounding by indication, and effectiveness and safety outcomes included for analysis. Of note, the PS-based methods are often not appropriately conducted, and thus sometimes affect the validity of the results[7]. Therefore, some studies used other estimators to further assess balance in covariates after the PS-based methods. We extracted the PS diagnostics (e.g., standardized difference [SD]) among the included studies.

Quality assessment

We assessed the quality of the eligible cohort studies based on the Newcastle- Ottawa Quality Scale (NOS). The NOS scale contains three domains with 9 points in total: the selection of the cohort (0-4 points), the comparability of the cohort (0-2 points) and the evaluations of the outcomes (0-3 points). Achieving ≥6 points indicates a moderate-to-high quality, whereas lower than 6 points represents a low quality[8]. Risk of bias assessment for randomized clinical trials was achieved by Cochrane assessment table[1].

Heterogeneity test and publication bias

The heterogeneity between the eligible studies were assessed through the Cochrane Q test and I^2 index. Significant heterogeneity was considered if the P value of the Cochrane Q test < 0.1 or if the I^2 value > 50%. The sources of heterogeneity were further estimated using subgroup analysis. We used the funnel plots to examine the publication bias for observational studies. Visual inspection of asymmetry indicates a bias. To further explore the potential bias statistically, the Begg's and Egger's tests were conducted.

Statistical analysis

We considered adjusted hazard ratios (HRs) and 95% confidence intervals (CIs) extracted from the eligible studies as the effect sizes. To calculate the overall effect size of an event outcome on a certain comparison (e.g., dabigatran versus warfarin), the logarithm of the HR ($Ln_{[HR]}$) and its corresponding standard error ($(ln_{[upper\ CI]}-ln_{[lower\ CI]})/3.92$) were conducted[9]. For each unique comparison outcome, DerSimonian and Laird random-effects (RE) model with an inverse variance method was used to pool the $Ln_{[HR]}$ due to its ability to draw a relatively conservative effect. However, the RE model provides wider CIs and may thus tolerate more statistical errors. Also, it shows a relatively high mean squared error, or makes unreasonable changes to individual study weights. Therefore, in the sensitivity analysis, we re-performed the meta-analysis using the fixed-effects model[8], inverse variance heterogeneity (IVhet)[10], and

quality effects (QE) models[11]. The subgroup analyses were conducted on the basis of drug dosage (low dose versus high dose), race (Asians versus non-Asians), age (≤75 versus > 75 years), follow-up period (≤12 versus > 12 months) and balance diagnostic methods (PSM or IPTW).

All the statistical analyses were carried out by Review Manager 5.3 software (the Cochrane Collaboration 2014. Nordic Cochrane Centre Copenhagen, Denmark), the Stata software (version 16.0, Stata Corp LP, College Station, TX) and MetaXL (version 5.3). A value of $P < 0.05$ was considered as statistically significant.

Supplementary Table 1. Baseline characteristics of the included phase III clinical trials (the whole population)

	RE-LY (Connolly et al. 2009)[4]			ROCKET-AF (Patel et al. 2011)[6]		ARISTOTLE (Granger et al. 2011)[3]		ENGAGE AF-TIMI 48 (Giugliano et al. 2013)[5]		
	Dabigatran, 110 mg	Dabigatran, 150 mg	Warfarin	Rivaroxaban, 20/15 mg	Warfarin	Apixaban, 5/2.5 mg	Warfarin	Edoxaban, 60/30 mg	Edoxaban 30/15 mg	Warfarin
**Basal characteristics**										
Sample size, n	6015	6076	6022	7131	7133	9120	9081	7035	7034	7036
Age, y	71.4	71.5	71.6	73.0	73.0	70.0	70.0	72.0	72.0	72.0
Female sex, %	35.7	36.8	36.7	39.7	39.7	35.5	35.0	37.9	38.8	37.5
Weight, kg	82.9	82.5	82.7	-	-	82	82	-	-	-
Body-mass index, kg/m2	-	-	-	28.3	28.1	-	-	-	-	-
CHADS2 (mean score)	2.1	2.2	2.1	3.5	3.5	2.1	2.1	2.8	2.8	2.8
**Comorbidities**										
Heart failure, %	32.2	31.8	31.9	62.6	62.3	35.5	35.4	58.2	56.6	57.5
Hypertension, %	78.8	78.9	78.9	90.3	90.8	87.3	87.6	93.7	93.5	93.6
Prior MI, %	16.8	16.9	16.1	16.6	18.0	14.5	13.9	-	-	-
Previous stroke/TIA, %	19.9	20.3	19.8	54.9	54.6	19.2	19.7	28.1	28.5	28.3
Peripheral vascular disease, %	-	-	-	5.6	6.1	-	-	-	-	-
Diabetes mellitus, %	23.4	23.1	23.4	40.4	39.5	25.0	24.9	36.4	36.2	35.8
**Medications**										
Aspirin, %	40.0	38.7	40.6	36.3	36.7	31.3	30.5	29.4	28.7	29.7
ARB or ACE inhibitors, %	66.3	66.7	65.5	-	-	70.9	70.1	-	-	-
Beta-blockers, %	62.9	63.7	61.8	-	-	63.6	62.6	-	-	-
Statin, %	44.9	43.9	44.4	-	-	45.0	45.1	-	-	-
Prior VKA, %	50.1	50.2	48.6	62.3	62.5	57.1	57.2	58.8	59.2	58.8
**TTR of warfarin users,%**	-	-	64.0	-	62.2	-	55.0	-	-	64.9
**INR targets for warfarin**	-	-	2.0-3.0	-	2.0-3.0	-	2.0-3.0	-	-	2.0-3.0

RE-LY= the Randomized Evaluation of Long-Term Anticoagulation Therapy; ROCKET-AF= the Rivaroxaban Once Daily Oral Direct Factor Xa Inhibition Compared with Vitamin K Antagonism for Prevention of Stroke and Embolism Trial in Atrial Fibrillation; ARISTOTLE= the Apixaban for Reduction in Stroke and Other Thromboembolic Events in Atrial Fibrillation; ENGAGE AF-TIMI 48= the Effective Anticoagulation with Factor Xa Next Generation in Atrial Fibrillation–Thrombolysis in Myocardial Infarction 48; $CHADS_2$=Congestive heart failure, Hypertension, 75 years of age and older, Diabetes mellitus, Stroke/transient ischemic attack history; MI=myocardial infarction; TIA=Transient ischemic attack; ARB= angiotensin receptor blocker; ACE= angiotensin converting enzyme; VKA=vitamin K antagonist; TTR=time within therapeutic range; INR=international normalized ratio.

Supplementary Table 2. Risk of bias assessment for randomized clinical trials

Included trails	Random sequence generation (selection bias)	Allocation concealment (selection bias)	Blinding of participants and personnel (performance bias)	Blinding of outcome assessment (detection bias)	Incomplete outcome data (attrition bias)	Selective reporting (reporting bias)	Other bias	Total
Connolly et al. 2009[4]	Low risk	Low risk	Low risk	Low risk	Low risk	Low risk	Low risk	Low
Granger et al. 2011[3]	Low risk	Low risk	Low risk	Low risk	Low risk	Low risk	Low risk	Low
Patel et al. 2011[6]	Low risk	Low risk	Low risk	Low risk	Low risk	Low risk	Low risk	Low
Giugliano et al. 2013[5]	Low risk	Low risk	Low risk	Low risk	Low risk	Low risk	Low risk	Low

Supplementary Table 3. Quality assessment of the included studies based on the NOS scale

Author	Selection				Comparability (maximum of two points)	Outcome			Total scores
	Representativeness of the exposed cohort	Selection of the non-exposed cohort	Ascertainment of exposure	Demonstration that outcome of interest was not present at start of study	Comparability of cohorts on the basis of the design or analysis	Assessment of outcome	Was follow-up long enough for outcomes to occur	Adequacy of follow up of cohorts	
Mitsuntisuk et al. 2020[12]	1	1	1	0	2	1	1	1	8
Nielsen et al. 2017[13]	1	1	1	0	2	1	1	1	8
Larsen et al. 2016[14]	1	1	1	0	2	1	1	1	8
Kohsaka et al. 2020[15]	1	1	1	0	2	1	1	1	8
Lee et al. 2019[16]	1	1	1	1	2	1	0	1	8
Cha et al. 2017[17]	1	1	1	1	2	1	1	1	9
Bang et al. 2020[18]	1	1	1	0	2	1	0	1	7
Chan et al. 2019[19]	1	1	1	0	2	1	1	1	8
Laliberte et al. 2014[20]	1	1	1	0	2	1	1	1	8
Wanat et al. 2019[21]	1	1	1	1	2	1	1	1	9
Gupta et al. 2019[22]	1	1	1	0	2	1	1	1	8
Villines et al. 2015[23]	1	1	1	0	2	1	1	1	8
Russo-Alvarez et al. 2018[24]	1	1	1	0	2	1	0	1	7
Adeboyeje et al. 2017[25]	1	1	1	0	2	1	1	1	8
Chang et al. 2015[26]	1	1	1	1	2	1	0	1	8
Lip et al. 2018[27]	1	1	1	0	2	1	1	1	8
Hernandez et al. 2015[28]	1	1	1	0	2	1	1	1	8
Huybrechts et al. 2019[29]	1	1	1	0	2	1	0	1	7
Bradley et al. 2020[30]	1	1	1	1	2	1	0	1	8
Go et al. 2017[31]	1	1	1	0	2	1	1	1	8

NOS=Newcastle-Ottawa Scale

Supplementary Table 4. Subgroup analysis of the primary and secondary outcomes between NOAC versus warfarin in patients

	Dabigatran vs Warfarin			Rivaroxaban vs Warfarin			Apixaban vs Warfarin			Edoxaban vs Warfarin		
	No. of effect estimates	HRs and 95% CIs	$P_{interaction}$	No. of effect estimates	HRs and 95% CIs	$P_{interaction}$	No. of effect estimates	HRs and 95% CIs	$P_{interaction}$	No. of effect estimates	HRs and 95% CIs	$P_{interaction}$
**SSE**												
Random-effects	9	0.82 (0.71,0.96)		10	0.80 (0.75,0.85)		10	0.75 (0.65,0.86)		2	0.71 (0.60,0.83)	
Fixed-effects	9	0.84 (0.79,0.89)	-	10	0.80 (0.76,0.84)	-	10	0.78 (0.75,0.81)	-	2	0.71 (0.60,0.83)	-
Dose												
Low dose*	3	0.94 (0.76,1.16)		3	0.83 (0.61,1.15)		5	0.71 (0.46,1.10)		-	-	
High dose*	5	0.82 (0.68,0.98)	0.32	4	0.77 (0.71,0.83)	0.61	5	0.64 (0.46,0.88)	0.70	2	0.76 (0.39,1.47)	-
Race												
Asians	4	0.70 (0.59,0.83)		4	0.75 (0.64,0.87)		4	0.63 (0.58,0.70)		2	0.71 (0.60,0.83)	
Non-Asians	5	0.93 (0.83,1.04)	0.007	6	0.82 (0.77,0.86)	0.29	6	0.82 (0.69,0.99)	0.01	-	-	-
Age												
≤75 y	7	0.79 (0.67,0.94)		6	0.81 (0.73,0.91)		6	0.76 (0.65,0.90)		-	-	
> 75 y	2	0.92 (0.71,1.19)	0.64	4	0.79 (0.73,0.85)	0.70	4	0.73 (0.54,0.99)	0.81	-	-	-
Follow-up period												
≤1 y	3	0.70 (0.54,0.89)		4	0.78 (0.73,0.84)		4	0.67 (0.55,0.82)		-	-	
> 1 y	5	0.90 (0.78,1.04)	0.08	5	0.80 (0.70,0.92)	0.74	5	0.82 (0.61,1.08)	0.27	2	0.71 (0.60,0.83)	-
Propensity score												
PSM	3	0.86 (0.75,0.98)		4	0.81 (0.76,0.87)		4	0.71 (0.58,0.86)		-	-	
IPTW	6	0.81 (0.65,1.02)	0.69	6	0.79 (0.70,0.88)	0.64	6	0.78 (0.60,1.00)	0.60	2	0.71 (0.60,0.83)	-
**MB**												
Random-effects	13	0.76 (0.65,0.87)		13	0.92 (0.84,1.00)		11	0.61 (0.56,0.67)		3	0.58 (0.40,0.74)	
Fixed-effects	13	0.78 (0.75,0.81)	-	13	1.01 (0.98,1.03)	-	11	0.61 (0.58,0.63)	-	3	0.60 (0.53,0.68)	-

Dose												
Low dose*	3	0.77 (0.53,1.10)		3	1.04 (0.78,1.39)		5	0.64 (0.45,0.90)		-	-	
High dose*	7	0.71 (0.65,0.79)	0.71	4	1.07 (1.02,1.12)	0.85	5	0.57 (0.49,0.66)	0.58	2	0.81 (0.15,4.39)	-
Race												
Asians	5	0.60 (0.50,0.73)		5	0.73 (0.65,0.82)		5	0.56 (0.47,0.67)		3	0.58 (0.40,0.74)	
Non-Asians	8	0.86 (0.72,1.02)	0.007	8	1.05 (1.02,1.08)	< 0.0001	6	0.64 (0.57,0.73)	0.21	-	-	-
Age												
≤75 y	10	0.71 (0.64,0.78)		9	0.87 (0.76,0.98)		7	0.56 (0.51,0.62)		2	0.51 (0.40,0.66)	
> 75 y	3	0.98 (0.62,1.56)	0.18	4	1.00 (0.86,1.18)	0.15	4	0.73 (0.59,0.89)	0.03	-	-	-
Follow-up period												
≤1 y	6	0.89 (0.68,1.17)		5	1.03 (0.96,1.10)		4	0.60 (0.57,0.63)		-	-	
> 1 y	5	0.63 (0.49,0.81)	0.06	5	0.81 (0.63,1.05)	0.08	5	0.61 (0.45,0.84)	0.87	2	0.56 (0.34,0.94)	-
Propensity score												
PSM	4	0.80 (0.71,0.90)		5	1.05 (1.02,1.08)		3	0.60 (0.57,0.63)		-	-	
IPTW	9	0.71 (0.56,0.90)	0.39	8	0.84 (0.73,0.98)	0.004	8	0.60 (0.51,0.71)	0.97	3	0.58 (0.40,0.74)	-

*Low dose refers to dabigatran 110 mg, rivaroxaban 15mg, apixaban 2.5mg and edoxaban 30mg; High dose refers to dabigatran 150mg, rivaroxaban 20mg, apixaban 5 mg and edoxaban 60mg.
NOAC= non-vitamin K antagonist oral anticoagulant; SSE=stroke or systemic embolism; MB=major bleeding; HR=hazard ratio; CI=confidence interval; y=year; PSM=propensity score matching; IPTW=inverse probability of treatment weighting.

Supplementary Table 5. Summary effect estimates of Egger's test and Begg's test for NOACs versus warfarin

	SSE	Major bleeding	Ischemic stroke	All-cause death	Intracranial hemorrhage	Gastrointestinal bleeding
**Dabigatran vs Warfarin**						
P value for Egger's test	0.742	0.749	0.396	0.165	0.010	0.201
P value for Begg's test	0.466	0.583	0.436	0.806	0.583	0.537
**Rivaroxaban vs Warfarin**						
P value for Egger's test	0.522	0.048	0.083	0.466	0.752	0.111
P value for Begg's test	0.474	0.428	0.152	0.734	0.436	0.721
**Apixaban vs Warfarin**						
P value for Egger's test	0.548	0.974	0.726	0.622	0.224	0.516
P value for Begg's test	0.858	0.876	1.000	1.000	0.938	0.917
**Edoxaban vs Warfarin**						
P value for Egger's test	-	0.593	0.505	-	-	0.328
P value for Begg's test	1.000	1.000	1.000	-	1.000	1.000

NOAC= non-vitamin K antagonist oral anticoagulant; SSE=stroke or systemic embolism.

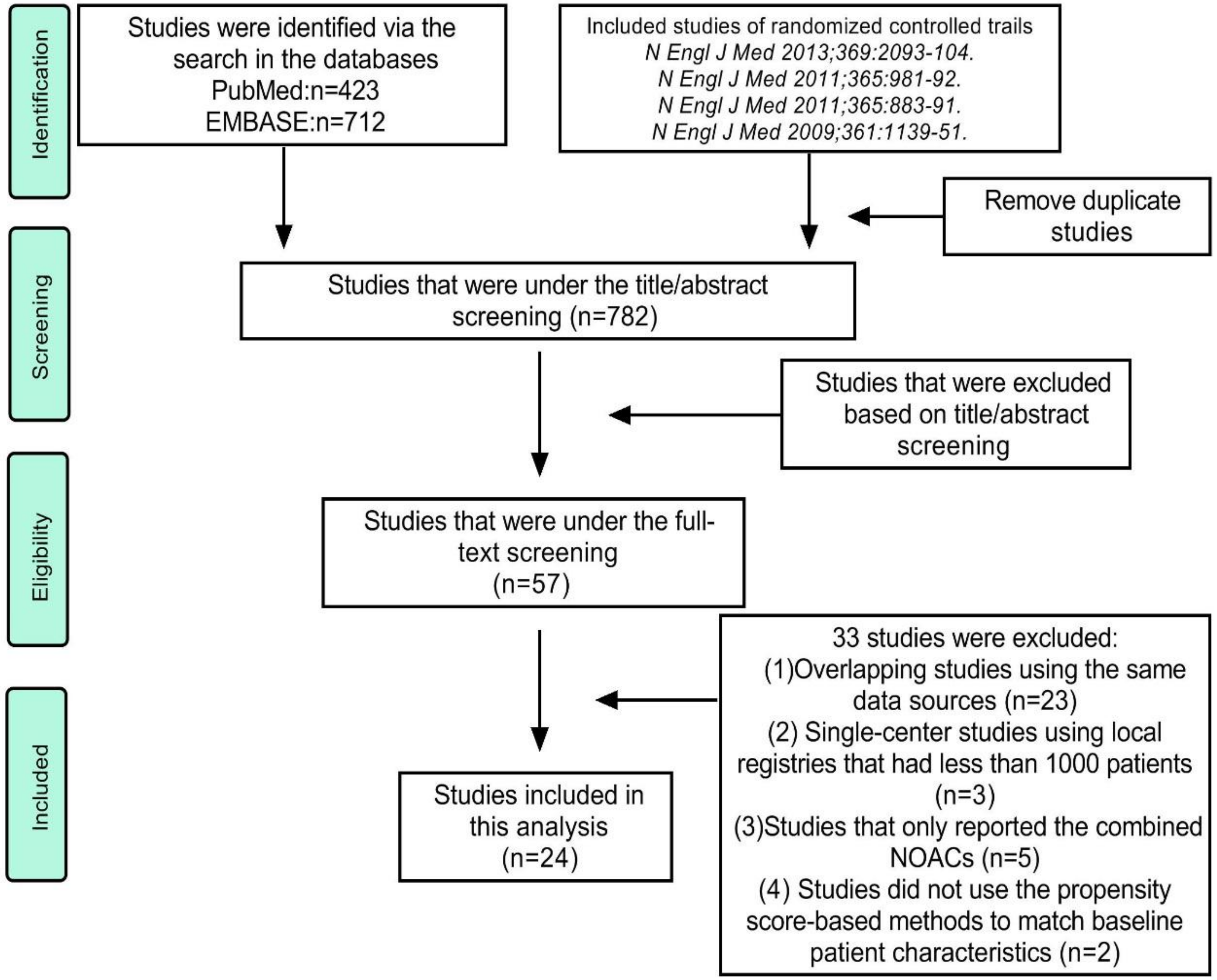

Supplementary Figure 1. Flow chart of document retrieval in this meta-analysis

N Engl J Med=New England Journal of Medicine

Study or Subgroup	log[Hazard Ratio]	SE	Weight	Hazard Ratio IV, Random, 95% CI
**4.1.1 SSE**				
Chan.et al. 2019	−0.4	0.163	27.7%	0.67 [0.49, 0.92]
Kohsaka et al. 2020	−0.329	0.101	72.3%	0.72 [0.59, 0.88]
**Subtotal (95% CI)**			**100.0%**	**0.71 [0.60, 0.83]**
Heterogeneity: $Tau^2 = 0.00$; $Chi^2 = 0.14$, df = 1 (P = 0.71); $I^2 = 0\%$				
Test for overall effect: Z = 4.06 (P < 0.0001)				
**4.1.2 IS**				
Chan.et al. 2019	−0.342	0.177	12.9%	0.71 [0.50, 1.00]
Kohsaka et al. 2020	−0.301	0.116	30.0%	0.74 [0.59, 0.93]
Lee et al. 2019	−0.464	0.084	57.2%	0.63 [0.53, 0.74]
**Subtotal (95% CI)**			**100.0%**	**0.67 [0.59, 0.76]**
Heterogeneity: $Tau^2 = 0.00$; $Chi^2 = 1.42$, df = 2 (P = 0.49); $I^2 = 0\%$				
Test for overall effect: Z = 6.29 (P < 0.00001)				
**4.1.3 Major bleeding**				
Chan.et al. 2019	−0.868	0.211	21.3%	0.42 [0.28, 0.63]
Kohsaka et al. 2020	−0.342	0.103	38.4%	0.71 [0.58, 0.87]
Lee et al. 2019	−0.587	0.093	40.3%	0.56 [0.46, 0.67]
**Subtotal (95% CI)**			**100.0%**	**0.58 [0.45, 0.74]**
Heterogeneity: $Tau^2 = 0.03$; $Chi^2 = 6.22$, df = 2 (P = 0.04); $I^2 = 68\%$				
Test for overall effect: Z = 4.35 (P < 0.0001)				
**4.1.4 ICH**				
Kohsaka et al. 2020	−0.083	0.079	51.9%	0.92 [0.79, 1.07]
Lee et al. 2019	−0.981	0.191	48.1%	0.37 [0.26, 0.55]
**Subtotal (95% CI)**			**100.0%**	**0.60 [0.25, 1.44]**
Heterogeneity: $Tau^2 = 0.38$; $Chi^2 = 18.88$, df = 1 (P < 0.0001); $I^2 = 95\%$				
Test for overall effect: Z = 1.15 (P = 0.25)				
**4.1.5 GIB**				
Chan.et al. 2019	−1.139	0.358	21.2%	0.32 [0.16, 0.65]
Kohsaka et al. 2020	−0.01	0.065	40.4%	0.99 [0.87, 1.12]
Lee et al. 2019	−0.462	0.106	38.4%	0.63 [0.51, 0.78]
**Subtotal (95% CI)**			**100.0%**	**0.65 [0.41, 1.04]**
Heterogeneity: $Tau^2 = 0.13$; $Chi^2 = 20.92$, df = 2 (P < 0.0001); $I^2 = 90\%$				
Test for overall effect: Z = 1.80 (P = 0.07)				

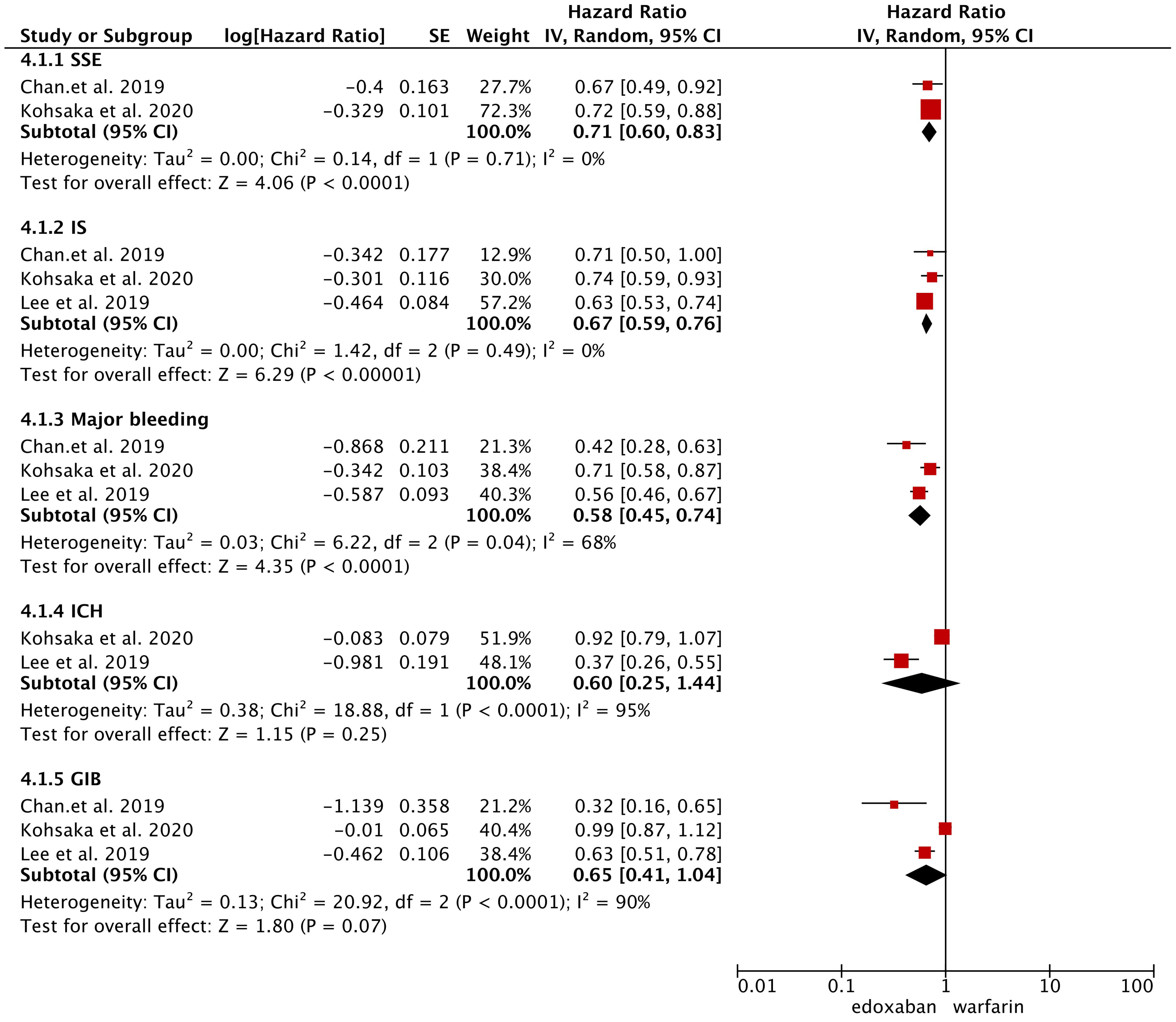

Supplementary Figure 2. Comparing the primary and secondary outcomes including SSE, MB, IS, all-cause death, ICH and GIB of edoxaban versus warfarin.

SSE= stroke or systemic embolism; MB= major bleeding; IS= ischemic stroke; ICH= intracranial haemorrhage; GIB= gastrointestinal bleeding; HR=hazard ratio; CI=confidence interval; SE=standard error; IV=inverse of the variance.

Study or Subgroup	log[Hazard Ratio]	SE	Weight	Hazard Ratio IV, Random, 95% CI
**1.2.1 IS**				
Cha et al. 2017	0.033	0.175	4.7%	1.03 [0.73, 1.46]
Chan.et al. 2019	−0.198	0.142	6.8%	0.82 [0.62, 1.08]
Go et al. 2017	−0.083	0.173	4.8%	0.92 [0.66, 1.29]
Gupta et al. 2019	−0.236	0.277	2.0%	0.79 [0.46, 1.36]
Huybrechts et al. 2020	−0.174	0.147	6.4%	0.84 [0.63, 1.12]
Kohsaka et al. 2020	−0.105	0.119	9.0%	0.90 [0.71, 1.14]
Larsen et al. 2016	0.058	0.119	9.0%	1.06 [0.84, 1.34]
Lee et al. 2019	−0.211	0.066	19.4%	0.81 [0.71, 0.92]
Lip et al. 2018	−0.01	0.085	14.5%	0.99 [0.84, 1.17]
Mitsuntisuk et al. 2020	−0.342	0.253	2.4%	0.71 [0.43, 1.17]
Nielsen et al. 2017	0.049	0.061	21.0%	1.05 [0.93, 1.18]
**Subtotal (95% CI)**			**100.0%**	**0.93 [0.86, 1.00]**
Heterogeneity: Tau² = 0.00; Chi² = 13.29, df = 10 (P = 0.21); I² = 25%				
Test for overall effect: Z = 1.88 (P = 0.06)				
**1.2.3 All-cause death**				
Cha et al. 2017	−0.654	0.146	22.8%	0.52 [0.39, 0.69]
Huybrechts et al. 2020	−0.186	0.133	23.5%	0.83 [0.64, 1.08]
Larsen et al. 2016	−0.431	0.097	25.2%	0.65 [0.54, 0.79]
Mitsuntisuk et al. 2020	−0.386	1.448	1.3%	0.68 [0.04, 11.61]
Nielsen et al. 2017	0.049	0.031	27.2%	1.05 [0.99, 1.12]
**Subtotal (95% CI)**			**100.0%**	**0.75 [0.53, 1.04]**
Heterogeneity: Tau² = 0.11; Chi² = 43.29, df = 4 (P < 0.00001); I² = 91%				
Test for overall effect: Z = 1.72 (P = 0.08)				
**1.2.4 ICH**				
Adeboyeje et al. 2017	−0.755	0.158	9.7%	0.47 [0.34, 0.64]
Bang et al. 2020	−0.616	0.163	9.6%	0.54 [0.39, 0.74]
Cha et al. 2017	−0.823	0.286	6.0%	0.44 [0.25, 0.77]
Chan.et al. 2019	−0.734	0.259	6.6%	0.48 [0.29, 0.80]
Go et al. 2017	−0.673	0.223	7.6%	0.51 [0.33, 0.79]
Gupta et al. 2019	−1.204	0.433	3.5%	0.30 [0.13, 0.70]
Hernandez et al. 2015	−1.139	0.234	7.3%	0.32 [0.20, 0.51]
Huybrechts et al. 2020	−0.942	0.219	7.7%	0.39 [0.25, 0.60]
Kohsaka et al. 2020	−0.236	0.093	11.9%	0.79 [0.66, 0.95]
Larsen et al. 2016	−0.942	0.186	8.8%	0.39 [0.27, 0.56]
Lee et al. 2019	−0.803	0.15	10.0%	0.45 [0.33, 0.60]
Lip et al. 2018	−0.844	0.13	10.7%	0.43 [0.33, 0.55]
Mitsuntisuk et al. 2020	−1.772	1.076	0.7%	0.17 [0.02, 1.40]
**Subtotal (95% CI)**			**100.0%**	**0.46 [0.38, 0.55]**
Heterogeneity: Tau² = 0.06; Chi² = 35.34, df = 12 (P = 0.0004); I² = 66%				
Test for overall effect: Z = 8.27 (P < 0.00001)				
**1.2.5 GIB**				
Adeboyeje et al. 2017	0.157	0.061	9.3%	1.17 [1.04, 1.32]
Bang et al. 2020	−0.186	0.095	8.8%	0.83 [0.69, 1.00]
Chan.et al. 2019	−0.248	0.225	6.4%	0.78 [0.50, 1.21]
Chang et al. 2015	0.191	0.119	8.4%	1.21 [0.96, 1.53]
Go et al. 2017	0.039	0.114	8.5%	1.04 [0.83, 1.30]
Gupta et al. 2019	0.231	0.174	7.4%	1.26 [0.90, 1.77]
Hernandez et al. 2015	0.615	0.059	9.3%	1.85 [1.65, 2.08]
Huybrechts et al. 2020	−0.117	0.077	9.1%	0.89 [0.76, 1.03]
Kohsaka et al. 2020	0.039	0.074	9.1%	1.04 [0.90, 1.20]
Lee et al. 2019	−0.361	0.089	8.9%	0.70 [0.59, 0.83]
Lip et al. 2018	−0.094	0.059	9.3%	0.91 [0.81, 1.02]
Mitsuntisuk et al. 2020	−1.139	0.28	5.4%	0.32 [0.18, 0.55]
**Subtotal (95% CI)**			**100.0%**	**0.97 [0.80, 1.17]**
Heterogeneity: Tau² = 0.10; Chi² = 154.48, df = 11 (P < 0.00001); I² = 93%				
Test for overall effect: Z = 0.34 (P = 0.73)				

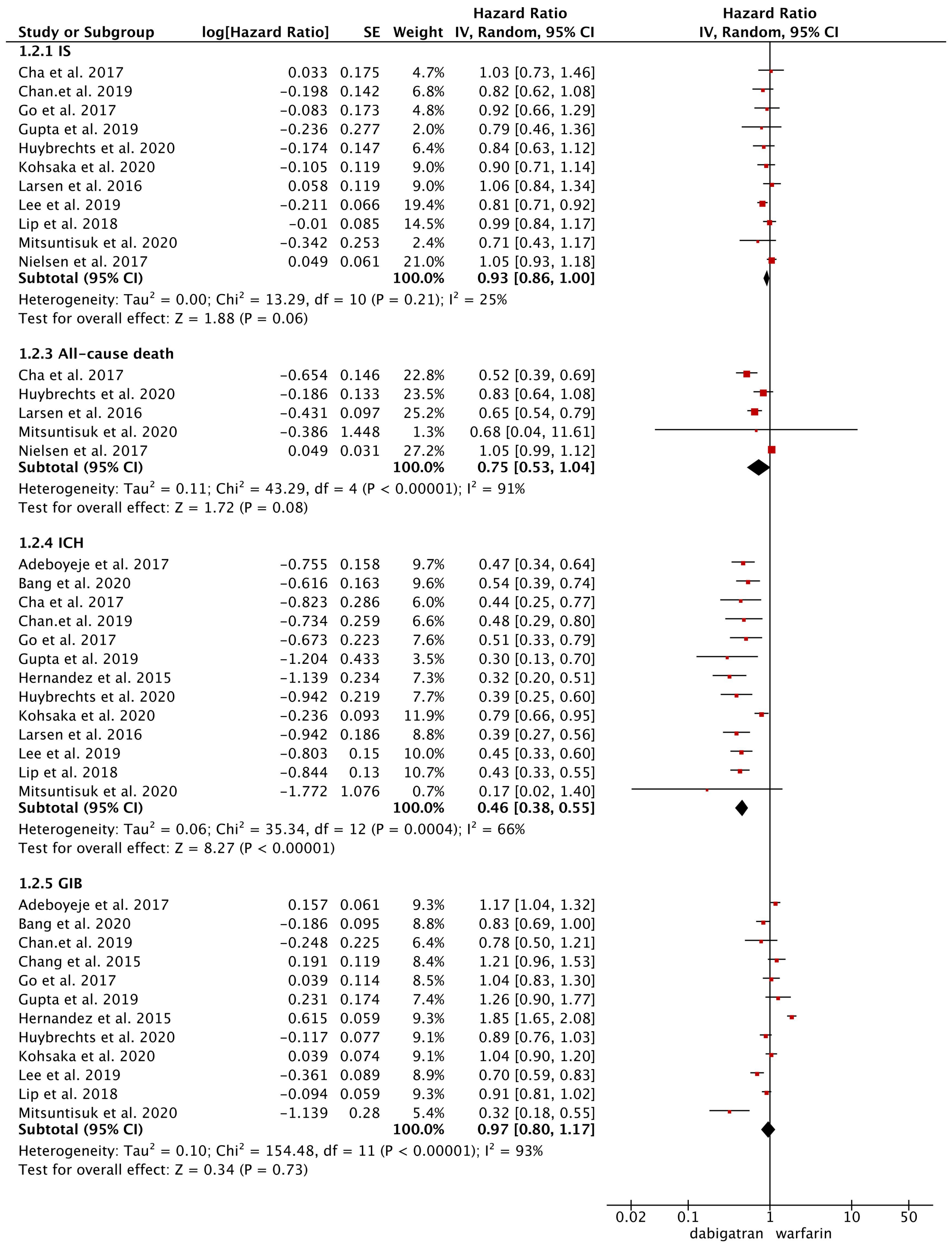

Supplementary Figure 3. Comparing the secondary outcomes including IS, all-cause death, ICH and GIB of dabigatran versus warfarin.

IS= ischemic stroke; ICH= intracranial haemorrhage; GIB= gastrointestinal bleeding; HR=hazard ratio; CI=confidence interval; SE=standard error; IV=inverse of the variance.

Study or Subgroup	log[Hazard Ratio]	SE	Weight	Hazard Ratio IV, Random, 95% CI
**2.2.1 IS**				
Cha et al. 2017	0.036	0.145	4.9%	1.04 [0.78, 1.38]
Chan.et al. 2019	−0.274	0.146	4.9%	0.76 [0.57, 1.01]
Gupta et al. 2019	0.113	0.162	4.1%	1.12 [0.82, 1.54]
Kohsaka et al. 2020	−0.301	0.103	8.8%	0.74 [0.60, 0.91]
Laliberte et al. 2014	−0.211	0.177	3.5%	0.81 [0.57, 1.15]
Larsen et al. 2016	−0.174	0.081	12.5%	0.84 [0.72, 0.98]
Lee et al. 2019	−0.233	0.034	29.2%	0.79 [0.74, 0.85]
Lip et al. 2018	−0.163	0.045	24.0%	0.85 [0.78, 0.93]
Mitsuntisuk et al. 2020	0.247	0.315	1.2%	1.28 [0.69, 2.37]
Nielsen et al. 2017	−0.051	0.118	7.0%	0.95 [0.75, 1.20]
**Subtotal (95% CI)**			**100.0%**	**0.84 [0.79, 0.90]**
Heterogeneity: $Tau^2 = 0.00$; $Chi^2 = 12.74$, df = 9 (P = 0.17); $I^2 = 29\%$				
Test for overall effect: Z = 4.95 (P < 0.00001)				
**2.2.2 All−cause death**				
Cha et al. 2017	−0.08	0.094	24.8%	0.92 [0.77, 1.11]
Larsen et al. 2016	−0.041	0.05	26.8%	0.96 [0.87, 1.06]
Mitsuntisuk et al. 2020	−0.236	0.149	21.5%	0.79 [0.59, 1.06]
Nielsen et al. 2017	0.385	0.048	26.9%	1.47 [1.34, 1.61]
**Subtotal (95% CI)**			**100.0%**	**1.02 [0.77, 1.36]**
Heterogeneity: $Tau^2 = 0.08$; $Chi^2 = 50.94$, df = 3 (P < 0.00001); $I^2 = 94\%$				
Test for overall effect: Z = 0.15 (P = 0.88)				
**2.2.3 ICH**				
Adeboyeje et al. 2017	−0.301	0.157	7.9%	0.74 [0.54, 1.01]
Bang et al. 2020	−0.416	0.136	9.9%	0.66 [0.51, 0.86]
Cha et al. 2017	−0.494	0.205	5.1%	0.61 [0.41, 0.91]
Chan.et al. 2019	−0.616	0.243	3.8%	0.54 [0.34, 0.87]
Gupta et al. 2019	−0.58	0.209	4.9%	0.56 [0.37, 0.84]
Kohsaka et al. 2020	−0.211	0.074	20.8%	0.81 [0.70, 0.94]
Laliberte et al. 2014	0.157	0.289	2.7%	1.17 [0.66, 2.06]
Larsen et al. 2016	−0.416	0.199	5.4%	0.66 [0.45, 0.97]
Lee et al. 2019	−0.334	0.103	14.5%	0.72 [0.59, 0.88]
Lip et al. 2018	−0.462	0.061	24.4%	0.63 [0.56, 0.71]
Mitsuntisuk et al. 2020	−0.868	0.651	0.6%	0.42 [0.12, 1.50]
**Subtotal (95% CI)**			**100.0%**	**0.69 [0.63, 0.76]**
Heterogeneity: $Tau^2 = 0.01$; $Chi^2 = 13.69$, df = 10 (P = 0.19); $I^2 = 27\%$				
Test for overall effect: Z = 7.46 (P < 0.00001)				
**2.2.4 GIB**				
Adeboyeje et al. 2017	0	0.073	12.0%	1.00 [0.87, 1.15]
Bang et al. 2020	−0.198	0.087	11.5%	0.82 [0.69, 0.97]
Chan.et al. 2019	−0.431	0.233	6.2%	0.65 [0.41, 1.03]
Chang et al. 2015	−0.02	0.513	2.0%	0.98 [0.36, 2.68]
Gupta et al. 2019	0.262	0.105	10.8%	1.30 [1.06, 1.60]
Kohsaka et al. 2020	−0.139	0.061	12.4%	0.87 [0.77, 0.98]
Laliberte et al. 2014	0.239	0.127	10.0%	1.27 [0.99, 1.63]
Lee et al. 2019	−0.219	0.07	12.1%	0.80 [0.70, 0.92]
Lip et al. 2018	0.215	0.029	13.1%	1.24 [1.17, 1.31]
Mitsuntisuk et al. 2020	−0.261	0.128	9.9%	0.77 [0.60, 0.99]
**Subtotal (95% CI)**			**100.0%**	**0.96 [0.82, 1.12]**
Heterogeneity: $Tau^2 = 0.05$; $Chi^2 = 80.63$, df = 9 (P < 0.00001); $I^2 = 89\%$				
Test for overall effect: Z = 0.49 (P = 0.62)				

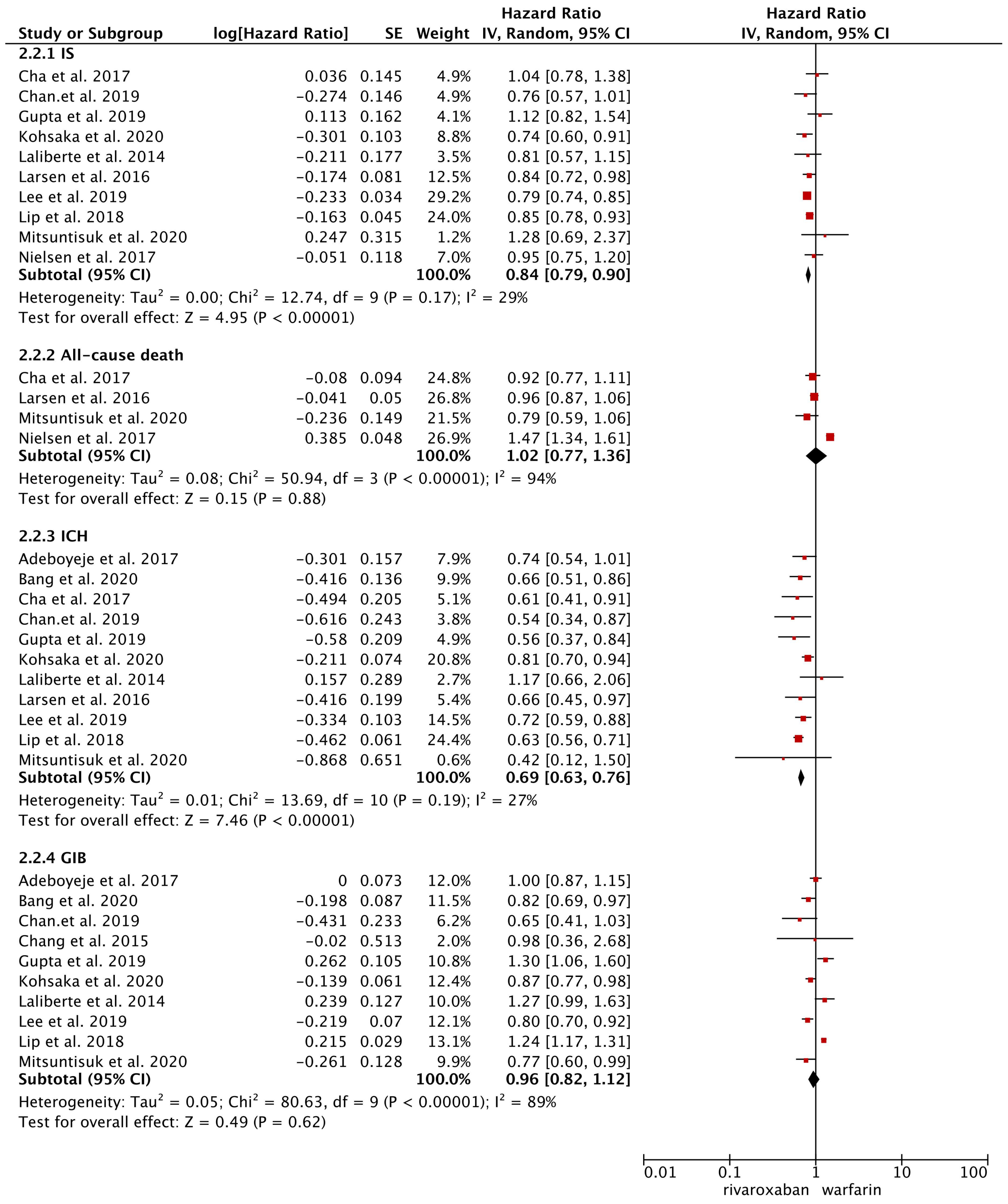

Supplementary Figure 4. Comparing the secondary outcomes including IS, all-cause death, ICH and GIB of rivaroxaban versus warfarin.

IS= ischemic stroke; ICH= intracranial haemorrhage; GIB= gastrointestinal bleeding; HR=hazard ratio; CI=confidence interval; SE=standard error; IV=inverse of the variance.

Study or Subgroup	log[Hazard Ratio]	SE	Weight	Hazard Ratio IV, Random, 95% CI
**3.2.1 IS**				
Bradley et al. 2020	−0.58	0.116	10.8%	0.56 [0.45, 0.70]
Cha et al. 2017	−0.396	0.31	4.7%	0.67 [0.37, 1.24]
Chan.et al. 2019	−0.342	0.154	9.3%	0.71 [0.53, 0.96]
Gupta et al. 2019	−0.386	0.209	7.4%	0.68 [0.45, 1.02]
Kohsaka et al. 2020	−0.462	0.095	11.6%	0.63 [0.52, 0.76]
Larsen et al. 2016	0.03	0.081	12.1%	1.03 [0.88, 1.21]
Lee et al. 2019	−0.378	0.066	12.6%	0.69 [0.60, 0.78]
Lip et al. 2018	−0.329	0.056	12.8%	0.72 [0.64, 0.80]
Mitsuntisuk et al. 2020	−0.734	0.203	7.6%	0.48 [0.32, 0.71]
Nielsen et al. 2017	0.207	0.107	11.1%	1.23 [1.00, 1.52]
**Subtotal (95% CI)**			**100.0%**	**0.73 [0.62, 0.86]**
Heterogeneity: $Tau^2 = 0.05$; $Chi^2 = 54.19$, df = 9 (P < 0.00001); $I^2 = 83\%$				
Test for overall effect: Z = 3.75 (P = 0.0002)				
**3.2.2 All-cause death**				
Cha et al. 2017	−1.13	0.282	26.6%	0.32 [0.19, 0.56]
Larsen et al. 2016	−0.4	0.072	31.6%	0.67 [0.58, 0.77]
Mitsuntisuk et al. 2020	0.285	0.918	10.1%	1.33 [0.22, 8.04]
Nielsen et al. 2017	0.438	0.057	31.8%	1.55 [1.39, 1.73]
**Subtotal (95% CI)**			**100.0%**	**0.77 [0.39, 1.54]**
Heterogeneity: $Tau^2 = 0.39$; $Chi^2 = 102.34$, df = 3 (P < 0.00001); $I^2 = 97\%$				
Test for overall effect: Z = 0.74 (P = 0.46)				
**3.2.3 ICH**				
Adeboyeje et al. 2017	−0.186	0.241	8.4%	0.83 [0.52, 1.33]
Bang et al. 2020	−0.994	0.292	6.9%	0.37 [0.21, 0.66]
Bradley et al. 2020	−0.635	0.143	12.1%	0.53 [0.40, 0.70]
Cha et al. 2017	−1.201	0.516	3.1%	0.30 [0.11, 0.83]
Chan.et al. 2019	−0.821	0.27	7.5%	0.44 [0.26, 0.75]
Gupta et al. 2019	−0.713	0.25	8.1%	0.49 [0.30, 0.80]
Kohsaka et al. 2020	−0.117	0.066	14.9%	0.89 [0.78, 1.01]
Larsen et al. 2016	−0.186	0.241	8.4%	0.83 [0.52, 1.33]
Lee et al. 2019	−0.395	0.121	13.0%	0.67 [0.53, 0.85]
Lip et al. 2018	−0.58	0.077	14.6%	0.56 [0.48, 0.65]
Mitsuntisuk et al. 2020	0.095	0.541	2.9%	1.10 [0.38, 3.18]
**Subtotal (95% CI)**			**100.0%**	**0.62 [0.50, 0.75]**
Heterogeneity: $Tau^2 = 0.07$; $Chi^2 = 39.28$, df = 10 (P < 0.0001); $I^2 = 75\%$				
Test for overall effect: Z = 4.76 (P < 0.00001)				
**3.2.4 GIB**				
Adeboyeje et al. 2017	−0.198	0.133	10.7%	0.82 [0.63, 1.06]
Bang et al. 2020	−0.511	0.102	12.3%	0.60 [0.49, 0.73]
Bradley et al. 2020	−0.562	0.071	13.8%	0.57 [0.50, 0.66]
Chan.et al. 2019	−1.427	0.348	3.8%	0.24 [0.12, 0.47]
Gupta et al. 2019	−0.342	0.131	10.8%	0.71 [0.55, 0.92]
Kohsaka et al. 2020	−0.139	0.056	14.5%	0.87 [0.78, 0.97]
Lee et al. 2019	−0.58	0.089	13.0%	0.56 [0.47, 0.67]
Lip et al. 2018	−0.511	0.043	14.9%	0.60 [0.55, 0.65]
Mitsuntisuk et al. 2020	−0.693	0.245	6.1%	0.50 [0.31, 0.81]
**Subtotal (95% CI)**			**100.0%**	**0.63 [0.54, 0.73]**
Heterogeneity: $Tau^2 = 0.04$; $Chi^2 = 50.33$, df = 8 (P < 0.00001); $I^2 = 84\%$				
Test for overall effect: Z = 6.00 (P < 0.00001)				

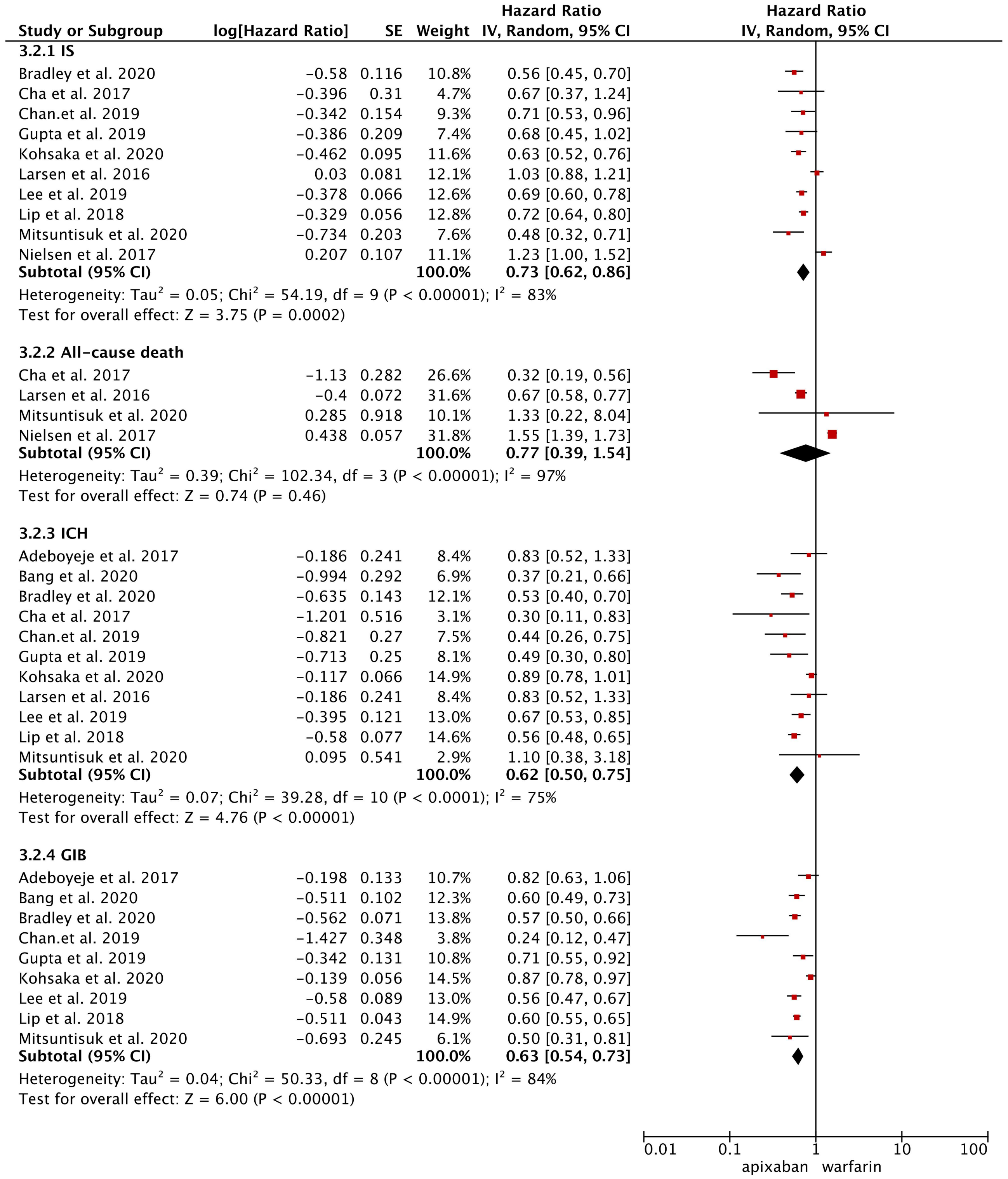

Supplementary Figure 5. Comparing the secondary outcomes including IS, all-cause death, ICH and GIB of apixaban versus warfarin.

IS= ischemic stroke; ICH= intracranial haemorrhage; GIB= gastrointestinal bleeding; HR=hazard ratio; CI=confidence interval; SE=standard error; IV=inverse of the variance.

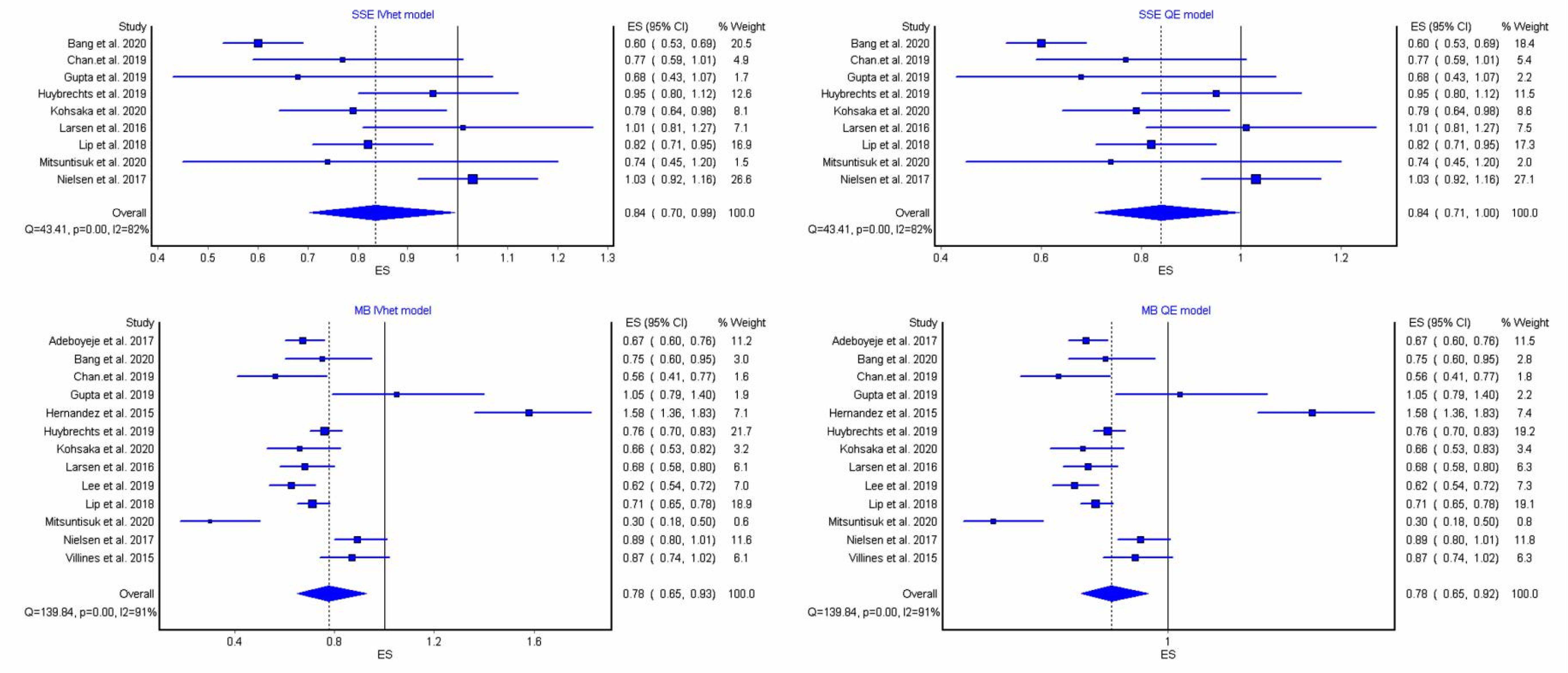

Supplementary Figure 6. Sensitivity analysis by using different models (Dabigatran vs Warfarin)

SSE=stroke or systemic embolism; MB=major bleeding; IVhet model= inverse variance heterogeneity model; QE model= quality effects model; ES=effect size; CI=confidence interval.

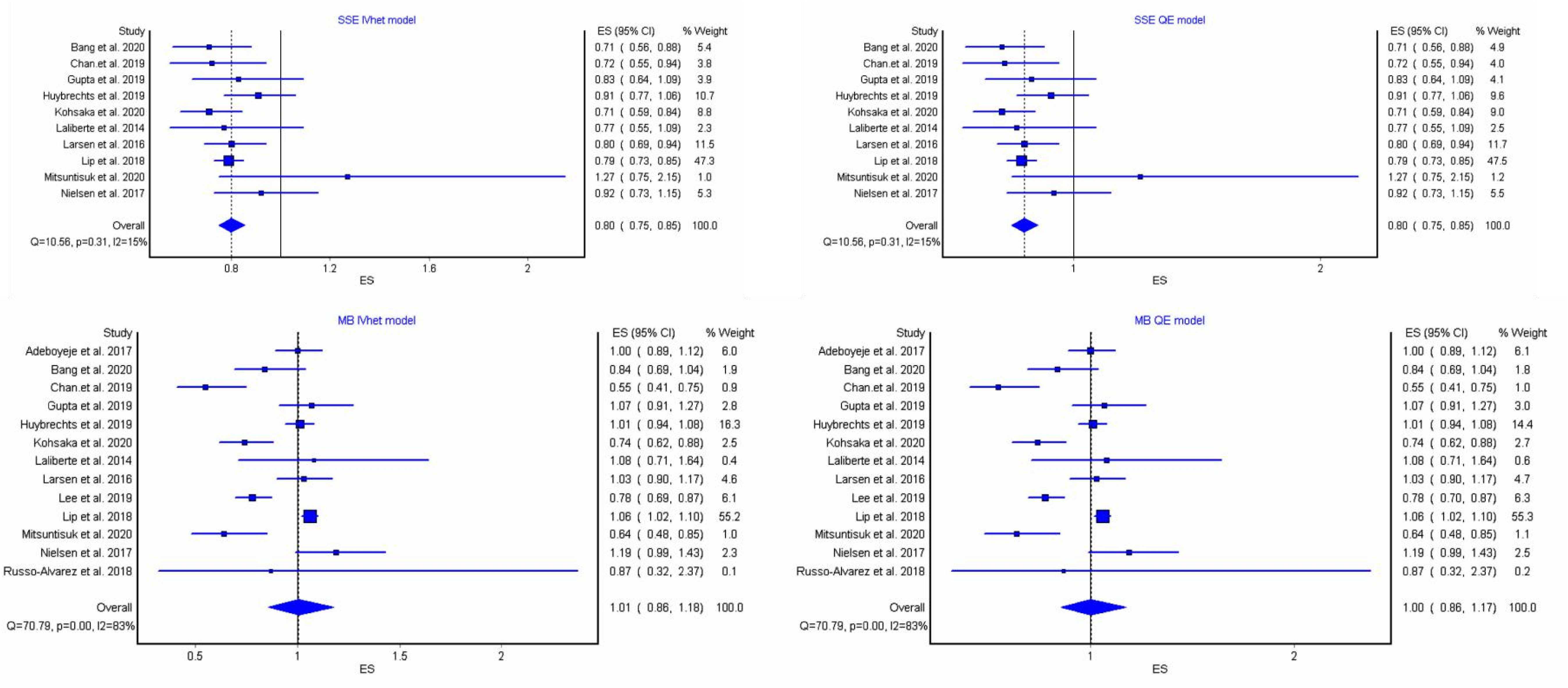

Supplementary Figure 7. Sensitivity analysis by using different models (Rivaroxaban vs Warfarin)

SSE=stroke or systemic embolism; MB=major bleeding; IVhet model= inverse variance heterogeneity model; QE model= quality effects model; ES=effect size; CI=confidence interval

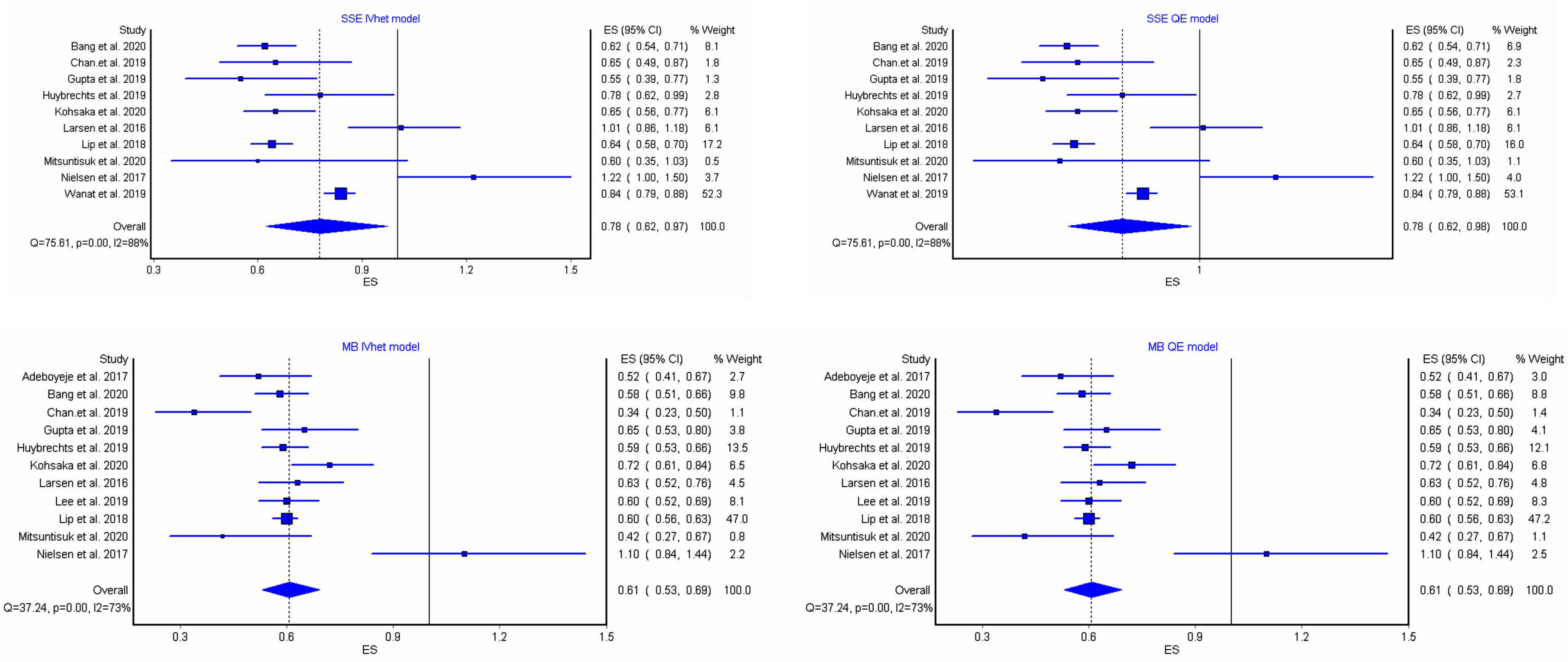

Supplementary Figure 8. Sensitivity analysis by using different models (Apixaban vs Warfarin)

SSE=stroke or systemic embolism; MB=major bleeding; IVhet model= inverse variance heterogeneity model; QE model= quality effects model; ES=effect size; CI=confidence interval

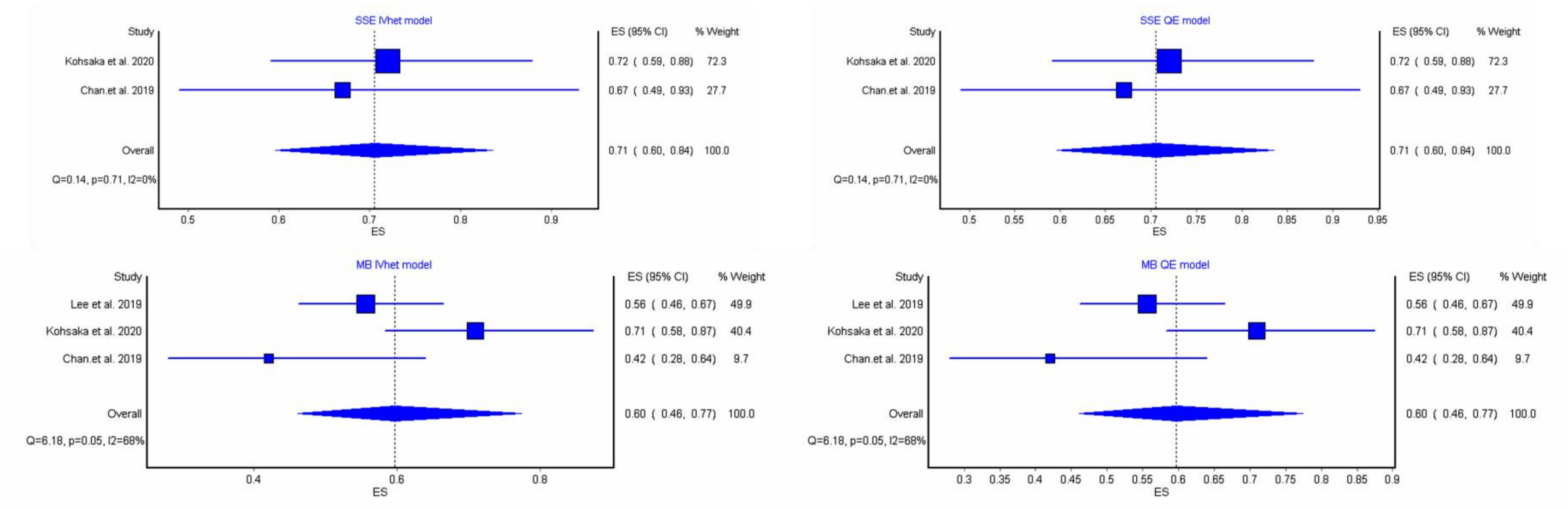

Supplementary Figure 9. Sensitivity analysis by using different models (Edoxaban vs Warfarin)

SSE=stroke or systemic embolism; MB=major bleeding; IVhet model= inverse variance heterogeneity model; QE model= quality effects model; ES=effect size; CI=confidence interval

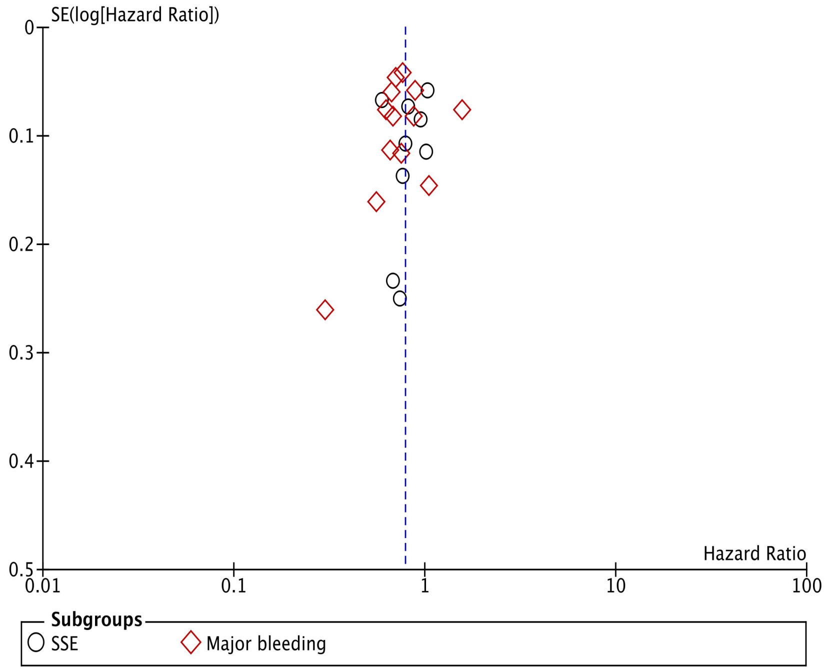

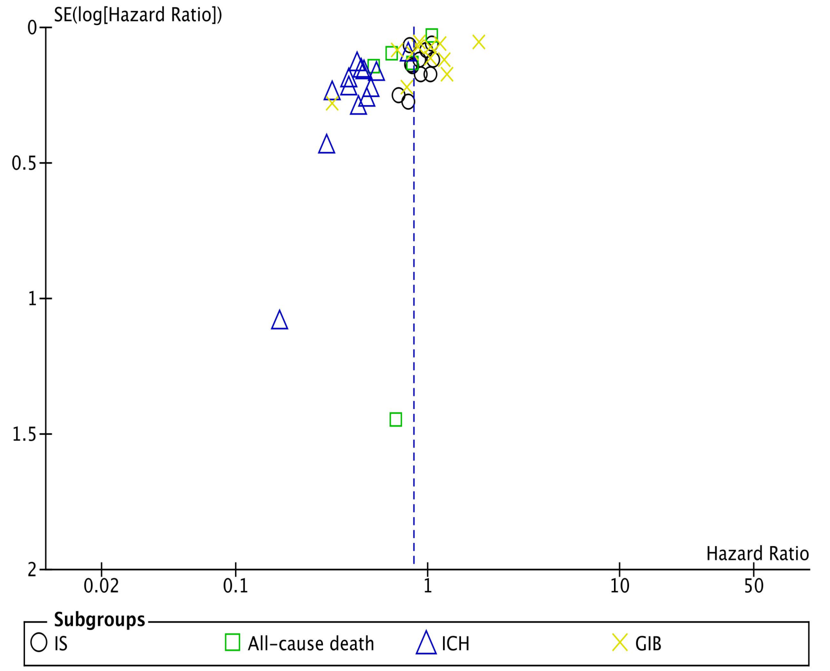

Supplementary Figure 10. Funnel plot for Dabigatran vs Warfarin

SSE=stroke or systemic embolism; IS=ischemic stroke; ICH=intracranial haemorrhage; GIB=gastrointestinal bleeding; SE=standard error.

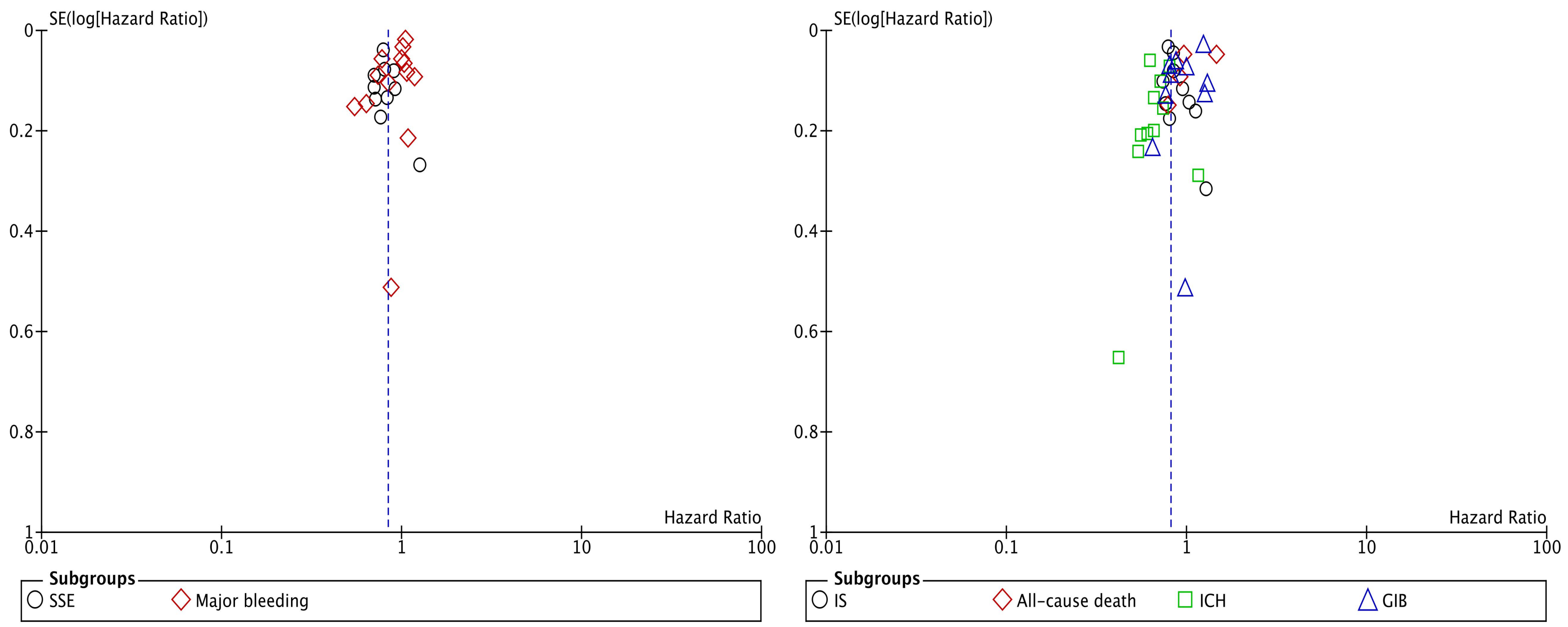

Supplementary Figure 11. Funnel plot for Rivaroxaban vs Warfarin

SSE=stroke or systemic embolism; IS=ischemic stroke; ICH=intracranial haemorrhage; GIB=gastrointestinal bleeding; SE=standard error.

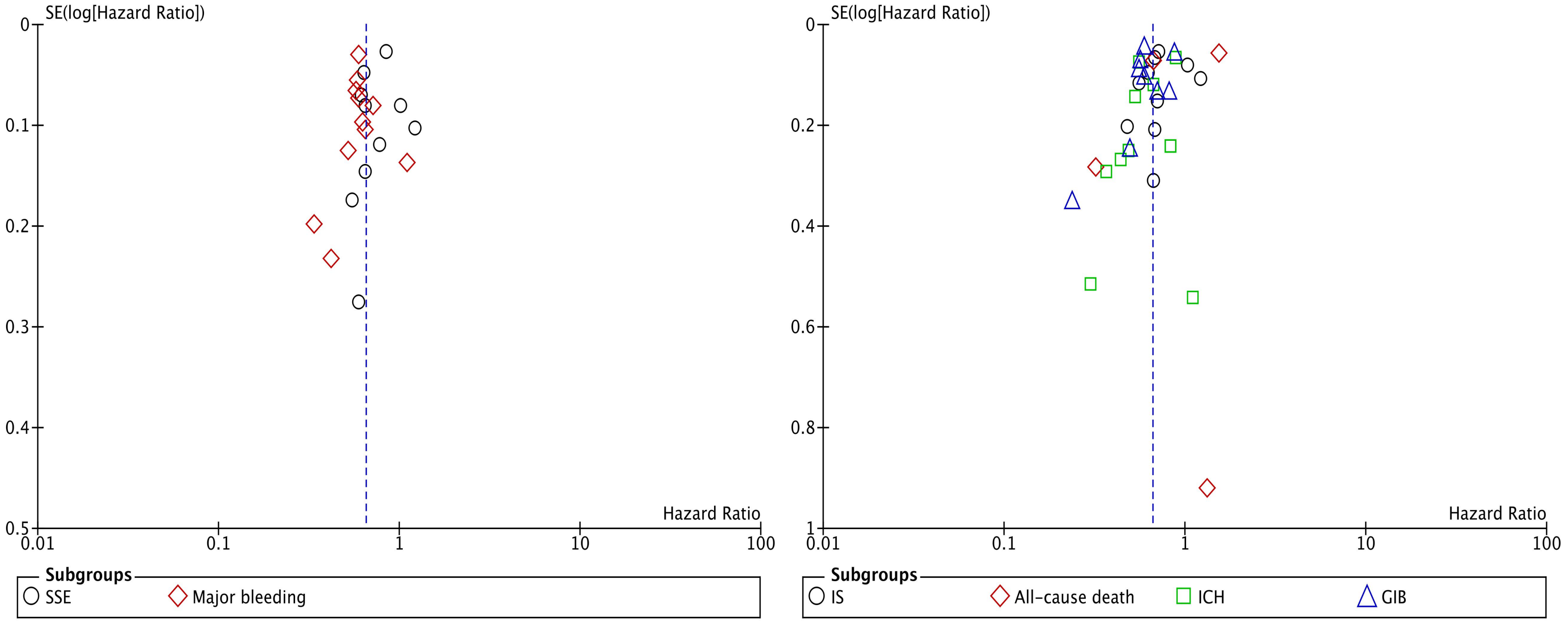

Supplementary Figure 12 Funnel plot for Apixaban vs Warfarin

SSE=stroke or systemic embolism; IS=ischemic stroke; ICH=intracranial haemorrhage; GIB=gastrointestinal bleeding; SE=standard error

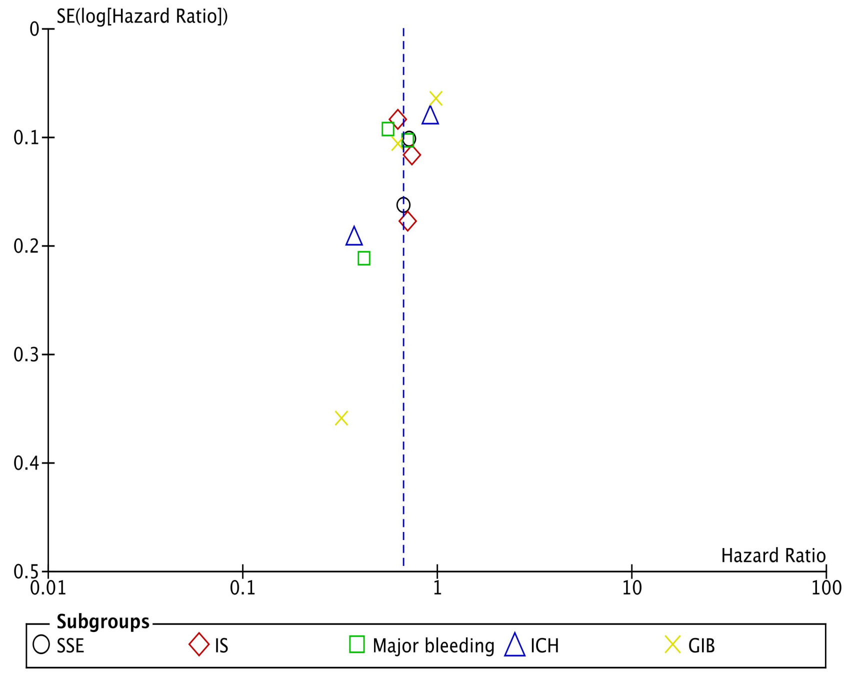

Supplementary Figure 13 Funnel plot for Edoxaban vs Warfarin

SSE=stroke or systemic embolism; IS=ischemic stroke; ICH=intracranial haemorrhage; GIB=gastrointestinal bleeding; SE=standard error

References:

1. Higgins J, Green S. Cochrane Handbook for Systematic Reviews of Interventions Version 5.1.0[updated March 2011]. Available at: www.cochrane-handbook.org. Accessed May 10, 2020.
2. Moher D, Liberati A, Tetzlaff J, Altman DG. Preferred reporting items for systematic reviews and meta-analyses: The PRISMA statement. *Plos Med*. 2009;6:e1000097
3. Granger CB, Alexander JH, McMurray JJ, Lopes RD, Hylek EM, Hanna M, Al-Khalidi HR, Ansell J, Atar D, Avezum A, Bahit MC, Diaz R, Easton JD, Ezekowitz JA, Flaker G, Garcia D, Geraldes M, Gersh BJ, Golitsyn S, Goto S, Hermosillo AG, Hohnloser SH, Horowitz J, Mohan P, Jansky P, Lewis BS, Lopez-Sendon JL, Pais P, Parkhomenko A, Verheugt FW, Zhu J, Wallentin L. Apixaban versus warfarin in patients with atrial fibrillation. *N Engl J Med*. 2011;365:981-992
4. Connolly SJ, Ezekowitz MD, Yusuf S, Eikelboom J, Oldgren J, Parekh A, Pogue J, Reilly PA, Themeles E, Varrone J, Wang S, Alings M, Xavier D, Zhu J, Diaz R, Lewis BS, Darius H, Diener HC, Joyner CD, Wallentin L. Dabigatran versus warfarin in patients with atrial fibrillation. *N Engl J Med*. 2009;361:1139-1151
5. Giugliano RP, Ruff CT, Braunwald E, Murphy SA, Wiviott SD, Halperin JL, Waldo AL, Ezekowitz MD, Weitz JI, Špinar J, Ruzyllo W, Ruda M, Koretsune Y, Betcher J, Shi M, Grip LT, Patel SP, Patel I, Hanyok JJ, Mercuri M, Antman EM. Edoxaban versus warfarin in patients with atrial fibrillation. *N Engl J Med*. 2013;369:2093-2104
6. Patel MR, Mahaffey KW, Garg J, Pan G, Singer DE, Hacke W, Breithardt G, Halperin JL, Hankey GJ, Piccini JP, Becker RC, Nessel CC, Paolini JF, Berkowitz SD, Fox KA, Califf RM. Rivaroxaban versus warfarin in nonvalvular atrial fibrillation. *N Engl J Med*. 2011;365:883-891
7. Zhang Z, Kim HJ, Lonjon G, Zhu Y. Balance diagnostics after propensity score matching. *Ann Transl Med*. 2019;7:16
8. Zhu W, Wan R, Liu F, Hu J, Huang L, Li J, Hong K. Relation of body mass index with adverse outcomes among patients with atrial fibrillation: A Meta-Analysis and systematic review. *J Am Heart Assoc*. 2016;5
9. Zhou Y, Ma J, Zhu W. Efficacy and safety of direct oral anticoagulants versus warfarin in patients with atrial fibrillation across BMI categories: A systematic review and Meta-Analysis. *Am J Cardiovasc Drugs*. 2020;20:51-60
10. Doi SA, Barendregt JJ, Khan S, Thalib L, Williams GM. Advances in the meta-analysis of heterogeneous clinical trials I: The inverse variance heterogeneity model. *Contemp Clin Trials*. 2015;45:130-138
11. Tadros HJ, Life CS, Garcia G, Pirozzi E, Jones EG, Datta S, Parvatiyar MS, Chase PB, Allen HD, Kim JJ, Pinto JR, Landstrom AP. Meta-analysis of cardiomyopathy-associated variants in troponin genes identifies loci and intragenic hot spots that are associated with worse clinical outcomes. *J Mol Cell Cardiol*. 2020;142:118-125
12. Mitsuntisuk P, Nathisuwan S, Junpanichjaroen A, Wongcharoen W, Phrommintikul A, Wattanaruengchai P, Rattanavipanon W, Chulavatnatol S, Chaiyakunapruk N, Likittanasombat K, Lip G. Real-World comparative effectiveness and safety of Non-Vitamin k antagonist oral anticoagulants vs. Warfarin in a developing country. *Clin Pharmacol Ther*. 2021;109:1282-1292
13. Nielsen PB, Skjøth F, Søgaard M, Kjældgaard JN, Lip GY, Larsen TB. Effectiveness and safety of reduced dose non-vitamin K antagonist oral anticoagulants and warfarin in patients with atrial fibrillation: Propensity weighted nationwide cohort study. *BMJ*. 2017;356:j510
14. Larsen TB, Skjøth F, Nielsen PB, Kjældgaard JN, Lip GY. Comparative effectiveness and safety of non-vitamin K antagonist oral anticoagulants and warfarin in patients with atrial fibrillation: Propensity weighted nationwide cohort study. *BMJ*. 2016;353:i3189
15. Kohsaka S, Katada J, Saito K, Jenkins A, Li B, Mardekian J, Terayama Y. Safety and effectiveness of non-vitamin K oral anticoagulants versus warfarin in real-world patients with non-valvular atrial fibrillation: A retrospective analysis of contemporary Japanese administrative claims data. *Open Heart*. 2020;7:e1232
16. Lee SR, Choi EK, Kwon S, Han KD, Jung JH, Cha MJ, Oh S, Lip G. Effectiveness and safety of contemporary oral anticoagulants among asians with nonvalvular atrial fibrillation. *Stroke*. 2019;50:2245-2249
17. Cha MJ, Choi EK, Han KD, Lee SR, Lim WH, Oh S, Lip G. Effectiveness and safety of Non-Vitamin k antagonist oral anticoagulants in asian patients with atrial fibrillation. *Stroke*. 2017;48:3040-3048
18. Bang OY, On YK, Lee MY, Jang SW, Han S, Han S, Won MM, Park YJ, Lee JM, Choi HY, Kang S, Suh HS, Kim YH. The risk of stroke/systemic embolism and major bleeding in Asian patients with non-valvular atrial fibrillation treated with non-vitamin K oral anticoagulants compared to warfarin: Results from a real-world data analysis. *Plos One*. 2020;15:e242922
19. Chan YH, Lee HF, See LC, Tu HT, Chao TF, Yeh YH, Wu LS, Kuo CT, Chang SH, Lip G. Effectiveness and safety of four direct oral anticoagulants in asian patients with nonvalvular atrial fibrillation. *Chest*. 2019;156:529-543
20. Laliberté F, Cloutier M, Nelson WW, Coleman CI, Pilon D, Olson WH, Damaraju CV, Schein JR, Lefebvre P. Real-world comparative effectiveness and safety of rivaroxaban and warfarin in nonvalvular atrial fibrillation patients. *Curr Med Res Opin*. 2014;30:1317-1325
21. Wanat MA, Wang X, Paranjpe R, Chen H, Johnson ML, Fleming ML, Abughosh SM. Warfarin vs. Apixaban in nonvalvular atrial fibrillation, and analysis by concomitant antiarrhythmic medication use: A national retrospective study. *Res Pract Thromb Haemost*. 2019;3:674-683
22. Gupta K, Trocio J, Keshishian A, Zhang Q, Dina O, Mardekian J, Nadkarni A, Shank TC. Effectiveness and safety of direct oral anticoagulants compared to warfarin in treatment naïve non-valvular atrial fibrillation patients in the US Department of defense population. *BMC Cardiovasc Disord*. 2019;19:142
23. Villines TC, Schnee J, Fraeman K, Siu K, Reynolds MW, Collins J, Schwartzman E. A comparison of the safety and effectiveness of dabigatran and warfarin in non-valvular atrial fibrillation patients in a large healthcare system. *Thromb Haemost*.

2015;114:1290-1298

24. Russo-Alvarez G, Martinez KA, Valente M, Bena J, Hu B, Luxenburg J, Chaitoff A, Ituarte C, Brateanu A, Rothberg MB. Thromboembolic and major bleeding events with rivaroxaban versus warfarin use in a Real-World setting. *Ann Pharmacother*. 2018;52:19-25

25. Adeboyeje G, Sylwestrzak G, Barron JJ, White J, Rosenberg A, Abarca J, Crawford G, Redberg R. Major bleeding risk during anticoagulation with warfarin, dabigatran, apixaban, or rivaroxaban in patients with nonvalvular atrial fibrillation. *J Manag Care Spec Pharm*. 2017;23:968-978

26. Chang HY, Zhou M, Tang W, Alexander GC, Singh S. Risk of gastrointestinal bleeding associated with oral anticoagulants: Population based retrospective cohort study. *BMJ*. 2015;350:h1585

27. Lip G, Keshishian A, Li X, Hamilton M, Masseria C, Gupta K, Luo X, Mardekian J, Friend K, Nadkarni A, Pan X, Baser O, Deitelzweig S. Effectiveness and safety of oral anticoagulants among nonvalvular atrial fibrillation patients. *Stroke*. 2018;49:2933-2944

28. Hernandez I, Baik SH, Piñera A, Zhang Y. Risk of bleeding with dabigatran in atrial fibrillation. *Jama Intern Med*. 2015;175:18-24

29. Huybrechts KF, Gopalakrishnan C, Bartels DB, Zint K, Gurusamy VK, Landon J, Schneeweiss S. Safety and effectiveness of dabigatran and other direct oral anticoagulants compared with warfarin in patients with atrial fibrillation. *Clin Pharmacol Ther*. 2020;107:1405-1419

30. Bradley M, Welch EC, Eworuke E, Graham DJ, Zhang R, Huang TY. Risk of stroke and bleeding in atrial fibrillation treated with apixaban compared with warfarin. *J Gen Intern Med*. 2020;35:3597-3604

31. Go AS, Singer DE, Toh S, Cheetham TC, Reichman ME, Graham DJ, Southworth MR, Zhang R, Izem R, Goulding MR, Houstoun M, Mott K, Sung SH, Gagne JJ. Outcomes of dabigatran and warfarin for atrial fibrillation in contemporary practice: A retrospective cohort study. *Ann Intern Med*. 2017;167:845-854
